# miRNA let-7 family regulated by NEAT1 and ARID3A/NF-κB inhibits PRRSV-2 replication *in vitro* and *in vivo*

**DOI:** 10.1371/journal.ppat.1010820

**Published:** 2022-10-10

**Authors:** Xiangbin You, Min Liu, Qian Liu, Huijuan Li, Yilin Qu, Xiaoxiao Gao, Chengyu Huang, Gan Luo, Gang Cao, Dequan Xu

**Affiliations:** 1 State Key Laboratory of Agricultural Microbiology, Huazhong Agricultural University, Wuhan, China; 2 Key Laboratory of Swine Genetics and Breeding of Ministry of Agriculture and Rural Affairs, Huazhong Agricultural University, Wuhan, China; 3 College of Animal Science & Technology, Huazhong Agricultural University, Wuhan, China; 4 College of Animal Science & Technology, Henan University of Science and Technology, Luoyang, China; 5 College of Veterinary Medicine, Huazhong Agricultural University, Wuhan, China; 6 Key Laboratory of Agricultural Animal Genetics, Breeding and Reproduction of Ministry of Education, Huazhong Agricultural University, Wuhan, China; University of Maryland School of Medicine, UNITED STATES

## Abstract

Porcine reproductive and respiratory syndrome (PRRS) is one of the most economically devastating diseases affecting the swine industry worldwide. To investigate the role of miRNAs in the infection and susceptibility of PRRS virus (PRRSV), twenty-four miRNA libraries were constructed and sequenced from PRRSV-infected and mock-infected Porcine alveolar macrophages (PAMs) of Meishan, Landrace, Pietrain and Qingping pigs at 9 hours post infection (hpi), 36 hpi, and 60 hpi. The let-7 family miRNAs were significantly differentially expressed between PRRSV-infected and mock-infected PAMs from 4 pig breeds. The let-7 family miRNAs could significantly inhibit PRRSV-2 replication by directly targeting the 3’UTR of the PRRSV-2 genome and porcine *IL6*, which plays an important role in PRRSV replication and lung injury. *NEAT1* acts as a competing endogenous lncRNA (ceRNA) to upregulate IL6 by attaching let-7 in PAMs. EMSA and ChIP results confirmed that ARID3A could bind to the promoter region of pri-let-7a/let-7f/let-7d gene cluster and inhibit the expression of the let-7 family. Moreover, the NF-κB signaling pathway inhibits the expression of the let-7 family by affecting the nuclear import of ARID3A. The pEGFP-N1-let-7 significantly reduced viral infections and pathological changes in PRRSV-infected piglets. Taken together, *NEAT1*/*ARID3A*/let-7/*IL6* play significant roles in PRRSV-2 infection and may be promising therapeutic targets for PRRS.

## Author summary

There are significant differences in susceptibility/resistance to PRRSV among different pig breeds. Especially the local pig breeds in China had strong resistance to PRRSV. However, due to the complexity of the interaction mechanism between pigs and PRRSV, the genetic mechanism leading to PRRSV susceptibility/resistance in different pig breeds is still unclear. MiRNAs play a vital regulatory role in immune response and development of PRRS. In this study, we found that the expression of miRNA let-7 family members were significantly different in PRRSV-infected/mock-infected PAMs from Pietrain, Qingping, Meishan, and Landrace pigs. Our findings illustrated that *NEAT1*/*ARID3A*/let-7/*IL6* had a significant role in PRRSV-2 infection. What’s more, let-7 family could significantly reduce PRRSV infection and pathological changes *in vitro* and *in vivo*. This discovery provided a new idea for breeding PRRSV resistant pigs by revealing the molecular mechanism of PRRSV susceptibility in different pig breeds. Altogether, let-7 family have significant roles in PRRSV infection and may be promising therapeutic targets for PRRS.

## Introduction

Porcine reproductive and respiratory syndrome (PRRS), also known as blue-ear pig disease, is one of the most economically devastating diseases affecting the swine industry worldwide [1]. It is characterized by high fever, high morbidity, high mortality, severe reproductive failure in pregnant sows and respiratory tract distress and persistent infection, particularly in suckling pigs [2]. PRRS virus (PRRSV) is a small enveloped single-stranded, 5’-capped positive sense RNA virus and is classified as a member of the family Arteriviridae, order *Nidovirales* [3]. Conventionally, all PRRSV isolates are classified into two genotypes: PRRSV-1(European-like isolate) and PRRSV-2(North America-like isolate) [4]. Porcine alveolar macrophages (PAMs) are immune defense cells of the lung that first contact pathogenic microorganisms [5] and are considered as the major target cells of viral replication during the acute infection period [6].

Some reports suggest that host genetics play a role in susceptibility to respiratory disease and differences in the severity and distribution of lesions in growing pigs caused by PRRSV [5,7–9]. For example, the Large White line had a significantly higher average percent monocyte-derived macrophages positive for PRRSV infection *in vitro* and *in vivo* than the Duroc-Pietrain synthetic line [10,11]. The Hampshire pigs had significantly more severe PRRSV-induced macroscopic lung lesions than did the Meishan or Duroc pigs [7]. Meishan pigs had significantly less PRRSV antigen detected in the lungs and significantly higher normalized serum antibody titers to PRRSV than Duroc pigs [12]. PRRS resistance and remarkable differences in susceptibility between breeds may be in part influenced by pig genetic factors.

MiRNAs play a vital regulatory role in the immune response and development of PRRS. Numerous eukaryotic miRNAs have been identified to be produced by hosts or viruses and target viral RNAs during infections. Many host miRNAs have been shown to affect diverse processes, including pathogenic diseases and host immunity [13]. For example, let-7 was initially described in C. elegans as a key gene involved in embryonic development and is a highly conserved miRNA in animal species [14]. The let-7 family of pigs includes let-7a, let-7c, let-7d, let-7e, let-7f, let-7g, let-7i and miR-98. More and more studies have shown that let-7 played a crucial role in the regulation of innate and adaptive immune responses, and was involved in the occurrence and development of viral diseases, cancer and other diseases [15,16]. Here, we showed that the let-7 family, which was significantly differentially expressed between PRRSV-infected and mock-infected PAMs from Pietrain, Qingping, Meishan, and Landrace pigs, had a significant inhibitory effect on PRRSV-2 replication *in vitro* and *in vivo*. In addition, the let-7 family was downregulated by *NEAT1* and *ARID3A*. The data showed that *NEAT1*/*ARID3A*/let-7/*IL6* had significant roles in PRRSV-2 infection and may be promising therapeutic targets for PRRS.

## Results

### miRNA-seq of PRRSV-infected/mock-infected PAMs from 4 pig breeds

**Fig 1:**
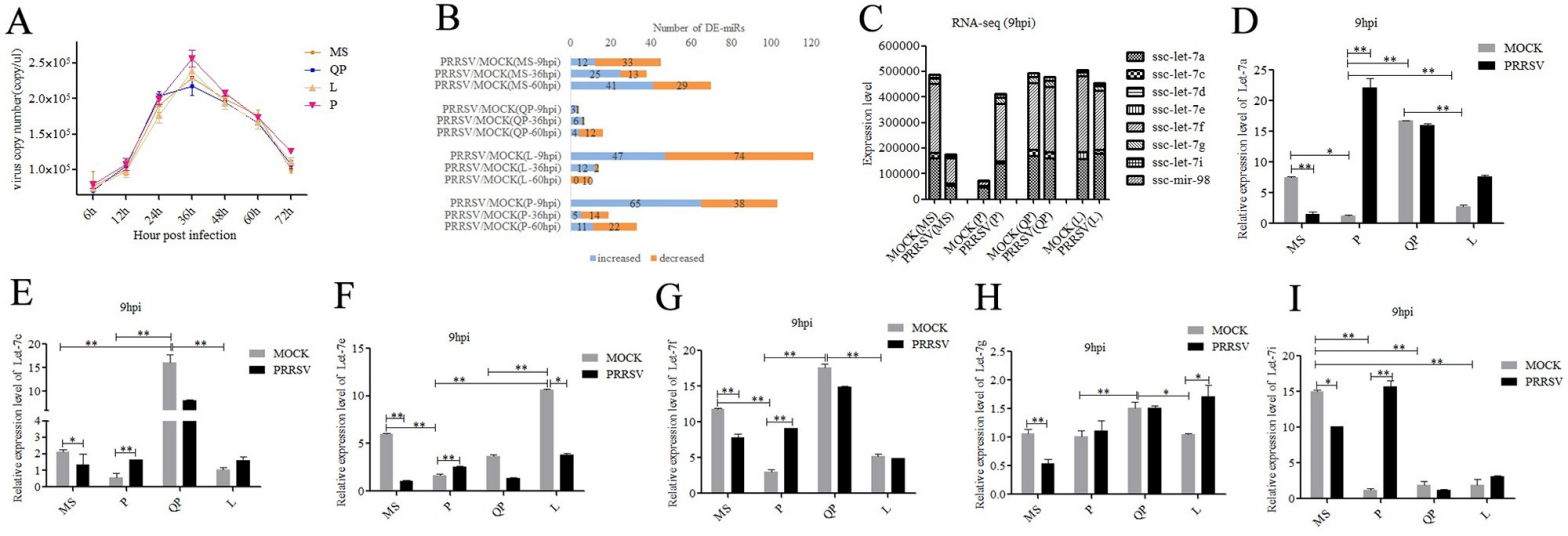
PRRSV-2 infection and DE-miRs analysis of PAMs from 4 pig breeds. PAMs were isolated from Meishan (MS), Qingping (QP), Pietrain (P), and Landrace(L) pigs and then infected with PRRSV-2 strains WuH3 (MOI = 0.1 PFU/cell). Cells were harvested at the indicated times, and the virus copy number was quantified by absolute quantitative PCR (A). The number of increased and decreased DE-miRs was analyzed between time-matched PRRSV-infected and mock-infected PAMs (B). Let-7 family expression levels were analyzed in miRNA-sequencing data of PRRSV-infected/mock-infected PAMs from different breeds of pigs at 9 hpi (C). The expression levels of miRNAs let-7a (D), let-7c (E), let-7e (F), let-7f (G), let-7g (H), and let-7i (I) were analyzed between PRRSV-infected and mock-infected PAMs from different pig breeds at 9 hpi by RT-qPCR. All the data for RT-qPCR were normalized to U6 and were represented as mean ± SD (n = 3). Statistical significance was analyzed by Student’s t test; *p<0.*05*, **p<0.*01*.

To understand the early, middle, and late stages of virus replication, the timing of cell harvesting was set at 6, 12, 24, 36, 48, and 72 hpi. As shown in Fig 1A, the curves continuously increased from 6 hpi to 36 hpi and continuously decreased from 36 hpi to 72 hpi in PAMs of all pig breeds. During 24–72 hpi, the virus copy number was different among the 4 pig breeds. The virus copy number in PAMs of Pietrain and Landrace pigs was higher than that of Qingping and Meishan pigs. According to virus growth curves, PAMs from the Meishan (MS), Landrace (L), Pietrain (P), and Qingping (QP) pig breeds infected with PRRSV for 9 h (early), 36 h (middle) and 60 h (later) were chosen to investigate the effect of PRRSV on PAM miRNAomes. As a control, mock-infected PAMs were collected at the same time (9 h, 36 h, and 60 h). Twenty-four miRNA libraries were constructed and analyzed using Solexa/Illumina deep-sequencing technology.

### The let-7 family was differentially expressed between PRRSV-infected and mock-infected PAMs from 4 pig breeds

Differentially expressed miRNAs (DE-miRs) were analyzed using previously reported methods [17–19]. As shown in Fig 1B, the DE-miRs were divided into increased and decreased expression groups. At 9 hpi, Pietrain and Landrace pigs had the largest number of DE-miRs, 121 and 103, respectively. Meanwhile, the total number of DE-miRs in Qingping pigs was the lowest. It seems that virus infection has the least effect on host immunity in Qingping pigs. At 36 hpi and 60 hpi, the number of DE-miRs in Meishan pigs was the largest, 38 and 70, respectively.

The let-7 family (ssc-let-7a, ssc-let-7c, ssc-let-7d, ssc-let-7e, ssc-let-7f, ssc-let-7g, ssc-let-7i and ssc-mir-98) was found to be significantly differentially expressed between PRRSV-infected and mock-infected PAMs (Fig 1C). The expression of the let-7 family was significantly lower in mock-infected PAMs from Pietrain pigs than in PAMs from the other three breeds. The results of stem–loop RT–qPCR were consistent with the sequencing results (Fig 1D–1I). In Meishan pigs, let-7 family expression was significantly decreased in PAMs infected with PRRSV-2 for 9 h, while let-7 family expression was significantly increased in PAMs from Pietrain pigs at 9 hpi.

### Let-7 family inhibits PRRSV-2 replication

**Fig 2:**
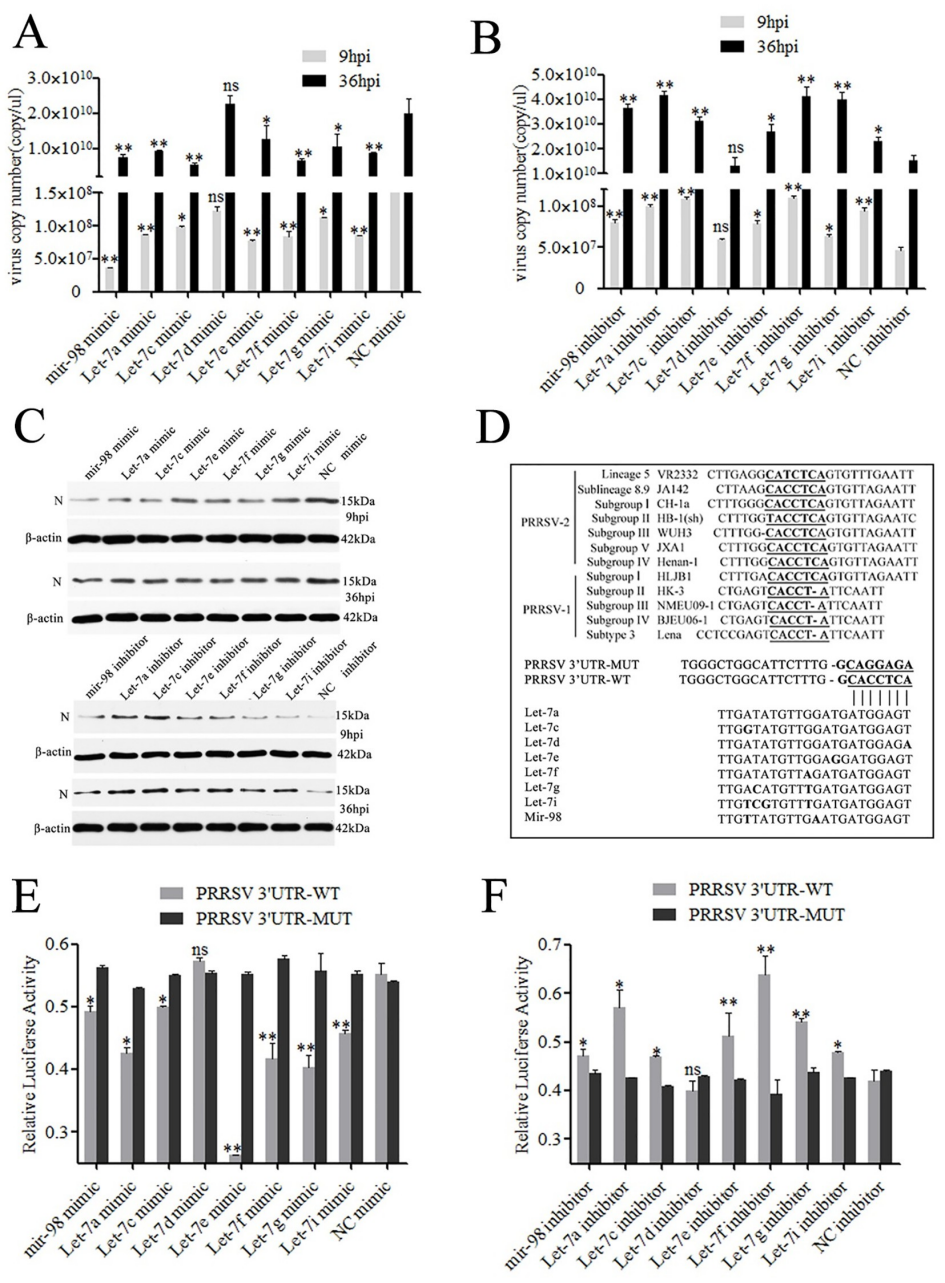
Let-7 family inhibits PRRSV-2 replication and directly target PRRSV-2 genomic RNA. Marc-145 cells were transfected with let-7 mimics/inhibitor, and then infected with PRRSV-2. The cells were collected at 9 hpi (9h) and 36 hpi (36h). The virus copy number was quantified by absolute quantitative PCR (A, B) and PRRSV-2 N protein was analyzed by Western blot (C). The let-7 binding site sequence in 3’UTR of 12 representative PRRSV-2 strains were predicted, compared and mutated (D). Luciferase activity was analyzed in Marc-145 cells co-transfected with PRRSV 3′ UTR or PRRSV 3′ UTR-MUT reporter constructs as indicated and either let-7 mimics (E) or let-7 inhibitor (F). Data are from three independent experiments (mean± SD). *p< 0.*05* and**p< 0.*01* (Student’s t test).

The mature sequences of let-7 family members of monkey and pig were completely consistent. Marc-145 cells, model cells for PRRSV studies, were transfected with the mimic or inhibitors of each let-7 family member (10 nM) and then infected with the PRRSV-2 strain WuH3 at an MOI of 0.1 and the cells were collected at 9 h and 36 h after infection. Compared with NC (negative control) mimics, mimics of the let-7 family, except for let-7d, significantly *(p<0*.*05)* or highly significantly *(p<0*.*01)* reduced the virus copy number at both 9 hpi and 36 hpi (Fig 2A). In contrast, inhibitors of the let-7 family facilitated PRRSV-2 replication. Meanwhile, the inhibitory effect of let-7a, let-7c, *l*et-7f, let-7i, and mir-98 on PRRSV-2 replication was significantly stronger than that of other let-7 family members (Fig 2B). Moreover, let-7 family members also reduced the expression of the PRRSV-2 N protein in the Western blot assay (Fig 2C).

### Let-7 family directly targets PRRSV-2 genomic RNA

Previous studies have shown that miRNAs might interact directly with the viral genome RNA to inhibit virus replication [20,21]. The prediction of miRNA target sites showed that the let-7 family could target the 3’UTR (15189 to 15195 bp) of the PRRSV-2 strain WuH3 genome. The 7-bp target region in PRRSV-2 is highly conserved. Furthermore, all members of the let-7 family share an almost identical 7-bp seed region, except let -7d is base A in the first base of the binding site (Fig 2D). Then, we created a reporter construct (PRRSV3’UTR-WT) containing the predicted 7-bp target site in the 3’UTR and another construct (PRRSV3’UTR-MUT) with mutations of four nucleotides in the 7-bp target site. The luciferase reporter assay results showed that let-7a, let-7c, let-7e, let-7f, let-7g, let-7i and mir-98 robustly downregulated the luciferase activity of PRRSV3’UTR-WT. Conversely, the luciferase activity of PRRSV3’UTR-MUT was not affected by the let-7 family (Fig 2E and 2F).

### *IL6* is also a direct target of the let-7 family

**Fig 3:**
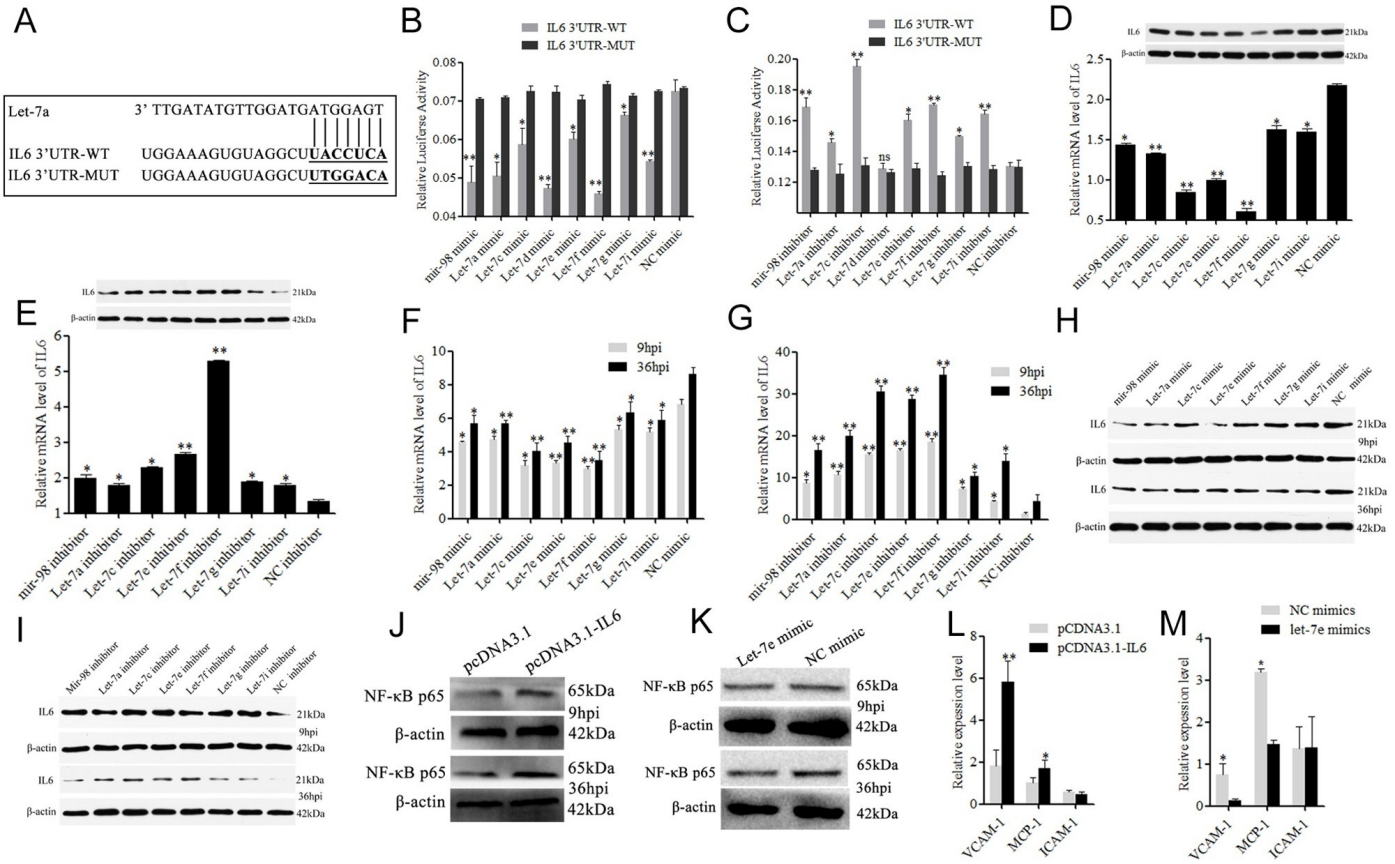
Let-7 family negatively regulate IL6 in Marc-145 cells. Bioinformatical predication showed that IL6 was a putative target gene of let-7 family (A). The dual luciferase reporter assay was performed in Marc-145 cells co-transfected with IL6 3’UTR-WT/IL6 3’UTR-MUT and let-7 mimics (B) or let-7 inhibitor (C). The expression of IL6 was analyzed in Marc-145 cells transfected with let-7 mimics (D) or inhibitor (E) by RT-qPCR and Western blot. Marc-145 cells were transfected with let-7 mimics/let-7 inhibitor, and then infected with PRRSV-2 for 9h or 36h, and the expressions of IL6 were detected by RT-qPCR (F, G) and Western blot (H, I). Marc-145 cells were transfected with pCDNA3.1-IL6 or let-7e mimics, and the expression of NF-κB p65 protein (J, K) and *VCAM-1*, *MCP-1*, and *ICAM-1* mRNA were detected (L, M). Data are expressed as the mean± standard deviation of three independent experiments. *P* values were calculated using a Student’s t test. *p<0.*05*, **p<0.*01*

In addition, miRNAs regulate viral replication by targeting host genes, which is also an important pathway. The let-7 family was predicted to target the porcine interleukin 6 (*IL6*) gene (Fig 3A). The mimics of the let-7 family significantly (*p <0*.*01* or *p <0*.*05*) inhibited the activity of the luciferase reporter plasmid containing the *IL6* 3’ UTR. Inhibitors of the let-7 family, except for let-7d, significantly (*p <0*.*01* or *p <0*.*05*) enhanced the luciferase activity. Moreover, the mimics and inhibitors of the let-7 family had no significant effect on the luciferase activity when transfected with the mutant plasmid IL6-MUT (Fig 3B and 3C). Compared with the NC group, the mimics of the let-7 family significantly (*p<0*.*01*) reduced *IL6* expression, and their inhibitors significantly (*p<0*.*05*) increased *IL6* expression at the mRNA and protein levels (Fig 3D and 3E). In particular, let-7f had the strongest effect. In addition, the let-7 family also repressed *IL6* mRNA and protein expression in PRRSV-infected Marc-145 cells (MOI = 0.1) at 9 hpi and 36 hpi (Fig 3F and 3H). The opposite results were observed in Marc-145 cells transfected with let-7 family inhibitors (Fig 3G and 3I).

### Let-7-*IL6* inhibits PRRSV-2 replication and attenuates lung injury via NF-κB and *MCP-1*/*VCAM-1*

*IL6* is a well-known pro-inflammatory factor, and its abnormally high expression can lead to tissue damage, and then affect the immune function of the host. The *IL6* gene in pigs and monkeys is highly conserved. So, an expression plasmid for porcine *IL6* (pCDNA3.1-IL6) was constructed and transfected into Marc-145 cells. The obtained results showed that pCDNA3.1-IL6 enhanced the expression of NF-κB p65 in Marc-145 cells infected with PRRSV-2 (Fig 3J). Let-7e mimics downregulated the expression of NF-κB p65 (Fig 3K). In addition, RT–qPCR results showed that *IL6* could significantly (*p<0*.*05*) influence the expression of macrophage cationic peptide 1 (*MCP-1*) and vascular cell adhesion molecule 1 (*VCAM-1*) (Fig 3L). Let-7e significantly (*p<0*.*05*) inhibited the expression of *MCP-1* and *VCAM-1* (Fig 3M), and play an important role in inflammatory diseases such as lung inflammation/injury.

### The lncRNA *NEAT1* and the let-7 family regulate each other in PAMs

**Fig 4:**
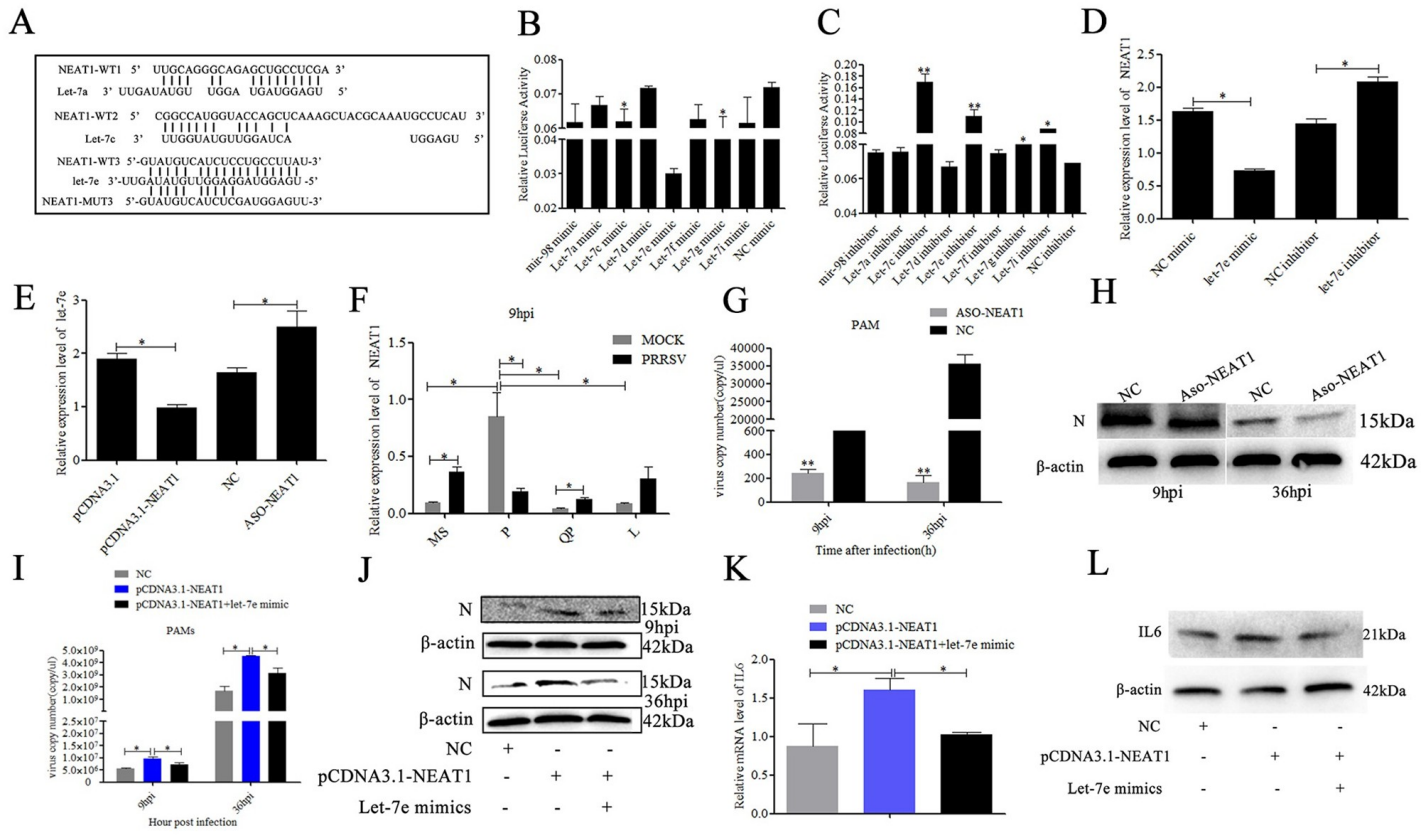
LncRNA *NEAT1* could inter-regulate with let-7 and PRRSV-2. The let-7 binding sites in *NEAT1* were predicted (A). Luciferase activity was analyzed in Marc-145 cells co-transfected with NEAT1-WT1, NEAT1-WT2 or NEAT1-WT3 reporter constructs as indicated and either corresponding Let-7 mimics (B) or Let-7 inhibitor (C). NEAT1 and let-7e regulate each other (D, E). Expression levels of *NEAT1* were analyzed between PRRSV-infected and mock-infected PAMs from 4 pig breeds at 9 hpi by RT-qPCR (F). PAMs were transfected with ASO-NEAT1, pcDNA3.1-NEAT1, pcDNA3.1-NEAT1+let-7e mimics or corresponding negative control (NC), and then infected with PRRSV (MOI = 0.1). The cells were harvested at 9 h and 36 h post PRRSV-2 infection. The virus copy number was quantified by absolute quantitative PCR (G, I) and PRRSV-2 N protein was analyzed by Western blot (H, J), and the expression of IL6 was detected by RT-qPCR (K) and Western blot (L). Three independent experiments were carried out. Error bars stand for the mean ± SD of at least triplicate experiments, *p<0.*05*, **p<0.*01*.

The ceRNA-miRNA-mRNA cross talk maintains the overall activity and functional balance of gene networks in a cell [22]. The lncRNA–miRNA–mRNA ceRNA network has been theorized to play an indispensable role in many types of diseases [23]. Bioinformatics analysis showed that let-7a, let-7d, let-7f, let-7i, and mir-98 target the 127–135 bp region of *NEAT1*, let-7c and let-7 g target the 1061–1069 bp region of *NEAT1*, and let-7e targets the 2612–2619 bp region of *NEAT1* (Fig 4A). The luciferase reporter plasmids NEAT1-WT1 (127–135 bp), NEAT1-WT2 (1061–1069 bp) and NEAT1-WT3 (2612–2619 bp) were constructed. The mimics of the let-7 family were transfected into Marc-145 cells with the corresponding luciferase reporter plasmid and the obtained results showed that let-7c, let-7e and let-7g significantly inhibited the luciferase activity (Fig 4B). Meanwhile, significantly increased (*p<0*.*01*) luciferase activity was observed when inhibitors of let-7c, let-7e, let-7g and let-7i were transfected with the corresponding luciferase reporter plasmid (Fig 4C). In addition, let-7e mimics had no significant effect (*p>0*.*05*) on the luciferase activity of NEAT1-MUT (S1 Fig). The RT–qPCR results showed that *NEAT1* expression was significantly elevated in the *let-7e* inhibitor group and decreased in the *let-7e* mimic group (Fig 4D). *Let-7e* expression was significantly downregulated in pCDNA3.1-NEAT1-transfected cells and upregulated in Aso-NEAT1 (Antisense oligonucleotide of NEAT1)-transfected cells (Fig 4E).

### *NEAT1* promotes PRRSV-2 replication through the let-7 family

*NEAT1* was differentially expressed and had an inverse difference from let-7 in different breeds of pigs and between PRRSV-infected and mock-infected PAMs (Fig 4F). To investigate whether NEAT1 regulate PRRSV-2 replication, PAMs were transfected with Aso-NEAT1, pCDNA3.1-NEAT1, pCDNA3.1-NEAT1 + let-7e mimics or matched negative control and infected with PRRSV-2 for 9 h and 36 h. The RT–qPCR and Western blot results showed that Aso-NEAT1 dramatically repressed PRRSV-2 replication (Fig 4G and 4H). On the other hand, overexpression of *NEAT1* could conspicuously promote PRRSV-2 replication. Let-7e mimics partially alleviated this trend (Fig 4I and 4J). Furthermore, *NEAT1* overexpression significantly increased *IL6* expression, while let-7e overexpression partly reduced *IL6* expression induced by *NEAT1* (Fig 4K and 4L).

### Identification of the promoter region and regulatory elements of the let-7a-2/let-7f-2/let-7d cluster

**Fig 5:**
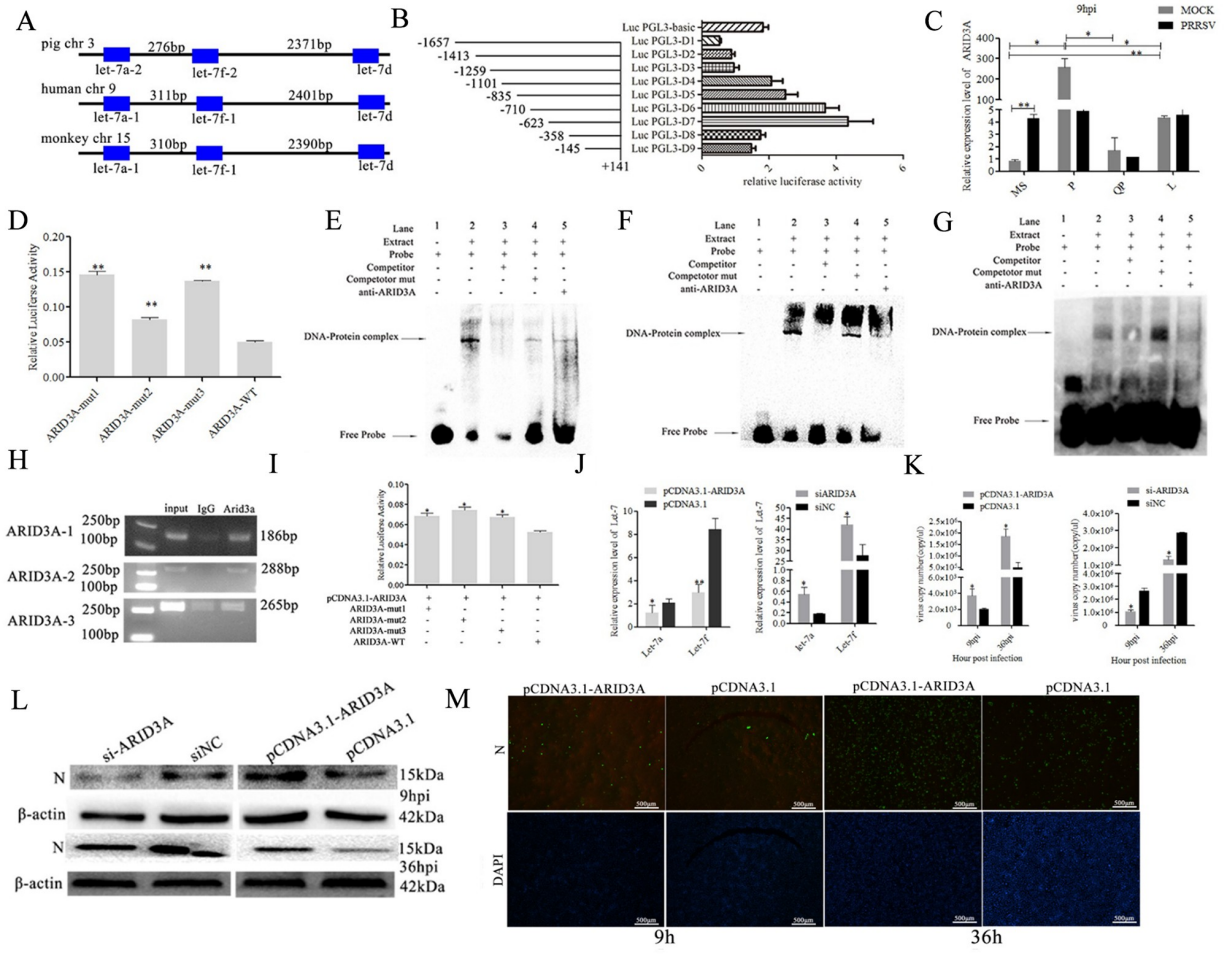
ARID3A binds to the promoters of let-7 and regulates let-7 expression and PRRSV-2 replication. Let-7a-2/let-7f-2/let-7d cluster is located on pig chromosome 3 (A). Let-7a-2/let-7f-2/let-7d promoter activity was identified by luciferase assay using truncated reporter constructs in Marc-145 cells (B). Expression levels of ARID3A were analyzed among PRRSV-infected/mock-infected PAMs from 4 pig breeds at 9 hpi by RT-qPCR (C). A mutation in the ARID3A binding site of let-7a-2/let-7f-2/let-7d promoter strongly increased transcriptional activity (D). The probes were incubated with nuclear extracts of PAMs in the absence or presence of a 50-fold excess of various competitor DNA oligos (mutant or unlabeled probes) or antibodies. The specific DNA–protein complex bands are indicated by arrows (E-G). ChIP assay results showed the interaction of ARID3A with the let-7a-2/let-7f-2/let-7d promoter *in vivo* in PAMs. DNA isolated from immunoprecipitated material was amplified by PCR for 35 cycles. Total chromatin was used as the input. Normal rabbit IgG was used as the negative control (H). The dual luciferase reporter assay was used to detect the luciferase activity in Marc-145 cells co-transfected with pcDNA3.1-ARID3A and ARID3A-mut1, ARID3A-mut2, ARID3A-mut3 or ARID3A-WT (I). Marc-145 cells were transfected with pCDNA3.1-ARID3A or siARID3A to detect the expression of let-7a/let-7f (J). Marc-145 cells were transfected with pCDNA3.1-ARID3A or siARID3A and then infected with PRRSV-2 to detect the replication of PRRSV-2 (K, L). Immunofluorescence assay of PRRSV-2 N protein was performed in Marc-145 cells transfected with pcDNA3.1-ARID3A or pcDNA3.1 and infected with PRRSV-2 for 9 h, 36 h (M). Three independent experiments were carried out. Error bars stand for the mean ± SD of at least triplicate experiments, *p<0.*05*, **p<0.*01*.

The precursor sequences of let-7a-2, let-7f-2 and let-7d come from the same miRNA cluster, which is located on pig chromosome 3 within a ~2647 bp region. A homologous let-7a-1/let-7f-1/let-7d cluster is found on human chromosome 9 and monkey chromosome 15 (Fig 5A). To investigate the transcriptional mechanism of let-7 family, the 1798 bp sequence on the 5´-flanking region of the let-7a-2/let-7f-2/let-7d cluster was obtained through PCR. A series of deletions of the pig let-7a-2/let-7f-2/let-7d potential promoter was used to drive luciferase gene expression and luciferase activity was determined in Marc-145 cells. The deletion of the region ranging from nucleotide -1657 to -623 in the PGL3-D7 (with the first nucleotide of pre-let-7a-2 assigned as +1) increased the promoter activation, which suggests that there are repressor binding sites in the deleted region. The results of further experiments with truncated let-7a-2/let-7f-2/let-7d promoters showed that the fragment from position -623 to position -358 was important for the activation of the let-7a-2/let-7f-2/let-7d promoter (Fig 5B).

### Transcription factor *ARID3A* binds to the promoter of let-7a-2/let-7f-2/let-7d both *in vivo* and *in vitro*

Three ARID3A transcription factor-binding sites were predicted in the -623 and -358 regions of the let-7a-2/let-7f-2/let-7d promoter. Furthermore, ARID3A binding sites were also predicted in the 2 kb region upstream of the other let-7 family member. *ARID3A* was significantly differentially expressed and had an inverse difference from let-7 between PRRSV-infected and mock-infected PAMs from 4 pig breeds (Fig 5C). Then, the three binding sites for ARID3A were mutated in the PGL3-D7 (-623 to +141) plasmid. The mutant plasmid ARID3A-mut-1, ARID3A-mut-2, ARID3A-mut-3.or the wide-type PGL3-D7 plasmid ARID3A-WT was respectively transfected into PAM. The results showed that luciferase activity of the three ARID3A-muts were significantly (*p<0*.*01*) higher than that of ARID3A-WT (Fig 5D). This indicated that *ARID3A* negatively regulated the expression of let-7a-2/let-7f-2/let-7d. Meanwhile, the EMSA was performed with nuclear extracts from PAMs to verify ARID3A binding to the promoter of let-7a-2/let-7f-2/let-7d. The specific DNA–protein complex was found in the biotin-labeled probe group containing the putative ARID3A binding sequences (Lane 2) and was attenuated after the addition of the competitor probe (Lane 3), while the mutated competitor probe failed to attenuate complex formation (Lane 4). The specific complex did not appear without nuclear extracts in Lane 1. Fig 5E, 5F and 5G respectively represent the three binding sites of ARID3A in let-7a-2/let-7f-2/let-7d promoters. Meanwhile, the ChIP assay was also performed. The immunoprecipitates were analyzed by PCR with primers specific to the let-7a-2/let-7f-2/let-7d promoter harboring the ARID3A binding site. These results clearly demonstrate that ARID3A binded the three sites in its promoter (Fig 5H).

### ARID3A regulates PRRSV-2 replication through let-7

To further determine whether ARID3A regulate let-7 expression, pCDNA3.1-ARID3A was cotransfected with PGL3-D7 (ARID3A-WT), ARID3A-mut-1, ARID3A-mut-2 or ARID3A-mut-3. The obtained results showed that the luciferase activity of the three ARID3A-mut plasmids was significantly (*p<0*.*05*) higher than that without mutation (Fig 5I). Furthermore, RT–qPCR results showed that overexpression of *ARID3A* significantly (*p<0*.*05*) suppressed the expression of let-7a and let-7f, whereas knockdown of *ARID3A* significantly (*p<0*.*05*) promoted (*p<0*.*05*) let-7a and let-7f expression (Fig 5J).

To determine whether ARID3A can affect PRRSV replication, pCDNA3.1-ARID3A, pCDNA3.1, si-ARID3A and si-NC were transfected into Marc-145 cells. The obtained results showed that overexpression of *ARID3A* significantly (*p<0*.*05*) promoted PRRSV-2 replication. Meanwhile, the opposite tendency was observed when cells were transfected with si-ARID3A (Fig 5K and 5L) (*p<0*.*05*). Furthermore, immunofluorescence assay (IFA) results showed that pCDNA3.1-ARID3A significantly promoted PRRSV-2 replication at 9 hpi and 36 hpi (Fig 5M).

### NF-κB p65 regulates let-7 expression through ARID3A

**Fig 6:**
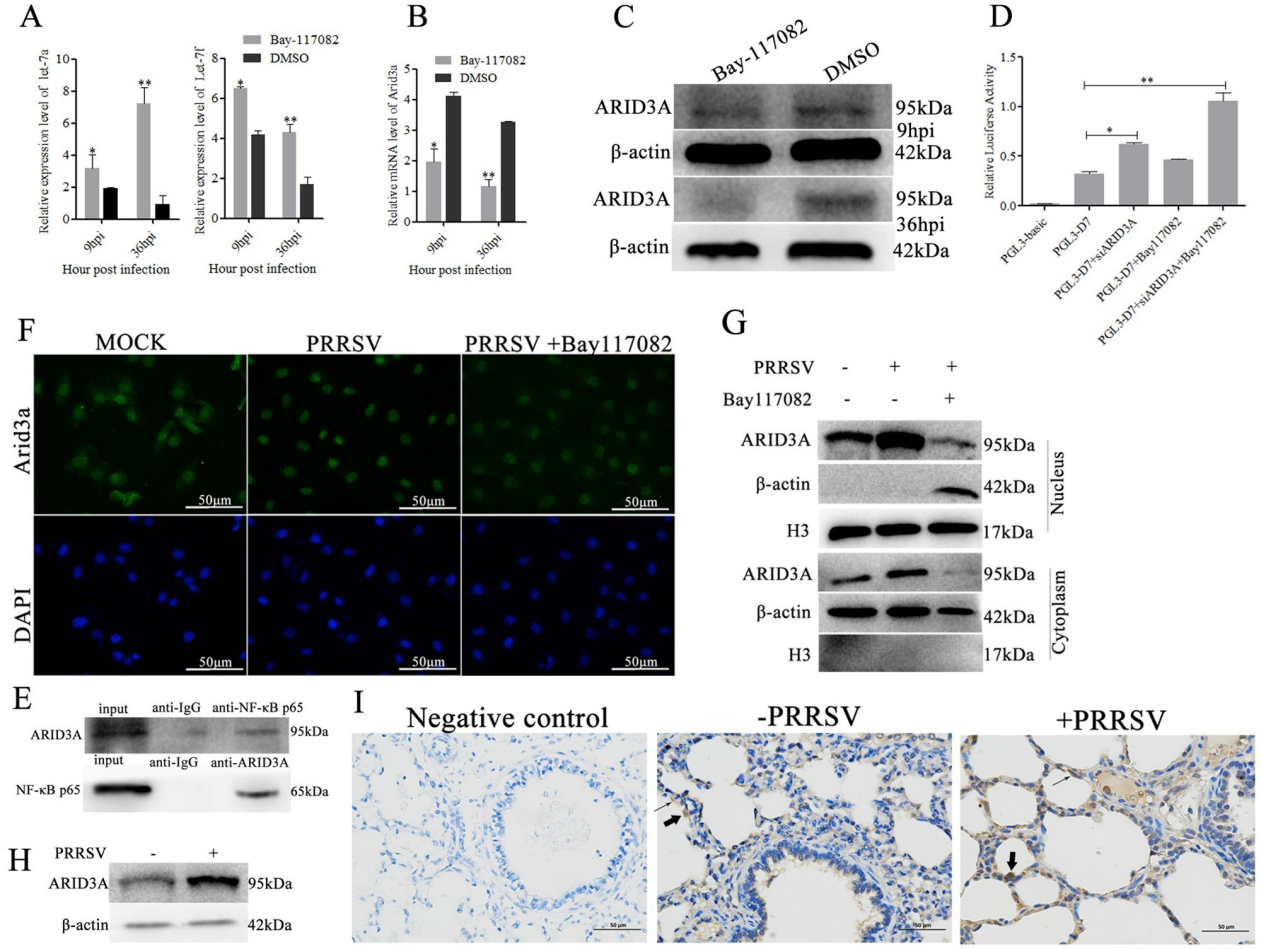
NF-κB regulates let-7 by ARID3A induced by PRRSV-2. Marc-145 cells were pretreatment with NF-kB inhibitor Bay117082, and infected with PRRSV-2 (MOI = 0.1) for 9h and 36h, and the expression of let-7a/let-7f (A) and ARID3A (B, C) were detected. Marc-145 cells were co-transfected with PGL3-D7 and si-ARID3A, Bay117082, or si-ARID3A+Bay117082, and the luciferase activity was analyzed (D). Co-IP assays were performed to investigate the interaction between ARID3A and NF-κB p65 with anti-ARID3A antibody, anti-NF-κB p65 antibody, and anti-IgG antibody as a negative control (E). The expression levels of ARID3A were measured by Western blot in the nucleus or cytoplasm of Marc-145 cells pretreated with Bay117082 and infected with PRRSV-2 for 36h (F). Immunofluorescence assays showed NF-κB p65 affecting nuclear import of ARID3A (G). The expression of ARID3A was analyzed in PRRSV-infected and mock-infected Marc-145 cells by Western blot (H). Immunohistochemistry staining (IHC) was used to analyze the cellular localization of ARID3A protein in PRRSV-infected and uninfected pig lungs. PAMs were labeled with thick arrows, while alveolar epithelial cells were labeled with thin arrows (I). Three independent experiments were carried out. Error bars stand for the mean ± SD of at least triplicate experiments, *p<0.*05*, **p<0.*01*.

To investigate how NF-κB p65 regulate let-7 expression, Marc-145 cells were pretreated with Bay117082 (5 mM), a specific NF-κB inhibitor, for 1 h prior to PRRSV-2 infection. The RT–qPCR results revealed that Bay117082 significantly promoted the expression of let-7a and let-7f at 9 hpi and 36 hpi (*p<0*.*01*) (Fig 6A). Meanwhile, ARID3A was significantly inhibited by Bay117082 (*p<0*.*01*) (Fig 6B and 6C). Then, the PGL3-D7 luciferase reporter plasmid was cotransfected with siARID3A, Bay117082 and siARID3A+Bay117082 into Marc-145 cells respectively. The luciferase activity was significantly activated by siARID3A and siARID3A+Bay117082 (Fig 6D). However, Bay117082 alone showed no significant (*p>0*.*05*) increase in PGL3-D7 activity. These results suggest that NF-κB p65 regulates let-7 family expression through the transcription factor ARID3A.

### NF-κB p65 interacts with ARID3A and promotes its nuclear import

To further investigate whether there is a direct interaction mechanism between ARID3A and NF-KB, coimmunoprecipitation (Co-IP) assays were performed in Marc-145 cells. The obtained results showed that ARID3A protein was detected in the immunoprecipitates of the anti-NF-κB p65 group, and NF-κB p65 protein was detected in the immunoprecipitates of the anti-ARID3A group, which demonstrated that ARID3A could interact with NF-κB p65 (Fig 6E). Meanwhile, immunofluorescence and Western blot showed that ARID3A was expressed in both the nucleus and cytoplasm when cells were in a normal state, increased in the nucleus after PRRSV infection, and was expressed in the nucleus and cytoplasm of cells after adding Bay117082 (Fig 6F and 6G). This finding indicated that NF-κB p65 promoted ARID3A nuclear import and regulation of the let-7 family. In addition, immunohistochemistry staining results showed that ARID3A was mainly located in alveolar epithelial cells and alveolar macrophages (Fig 6I). Meanwhile, the protein expression of ARID3A was significantly increased in Marc-145 cells after PRRSV-2 infection (Fig 6H).

### Let-7 family inhibits PRRSV-2 replication in piglets

To study the role of the let-7 family in pigs infected with PRRSV-2, the recombinant plasmid pEGFP-N1-let-7, which containing all eight let-7 family members coexpressing simultaneously, was constructed (S2A Fig). The obtained results showed that pEGFP-N1-let-7 could significantly (*p<0*.*001*) inhibit PRRSV replication and IL6 expression (S2B–S2F Fig).

**Fig 7:**
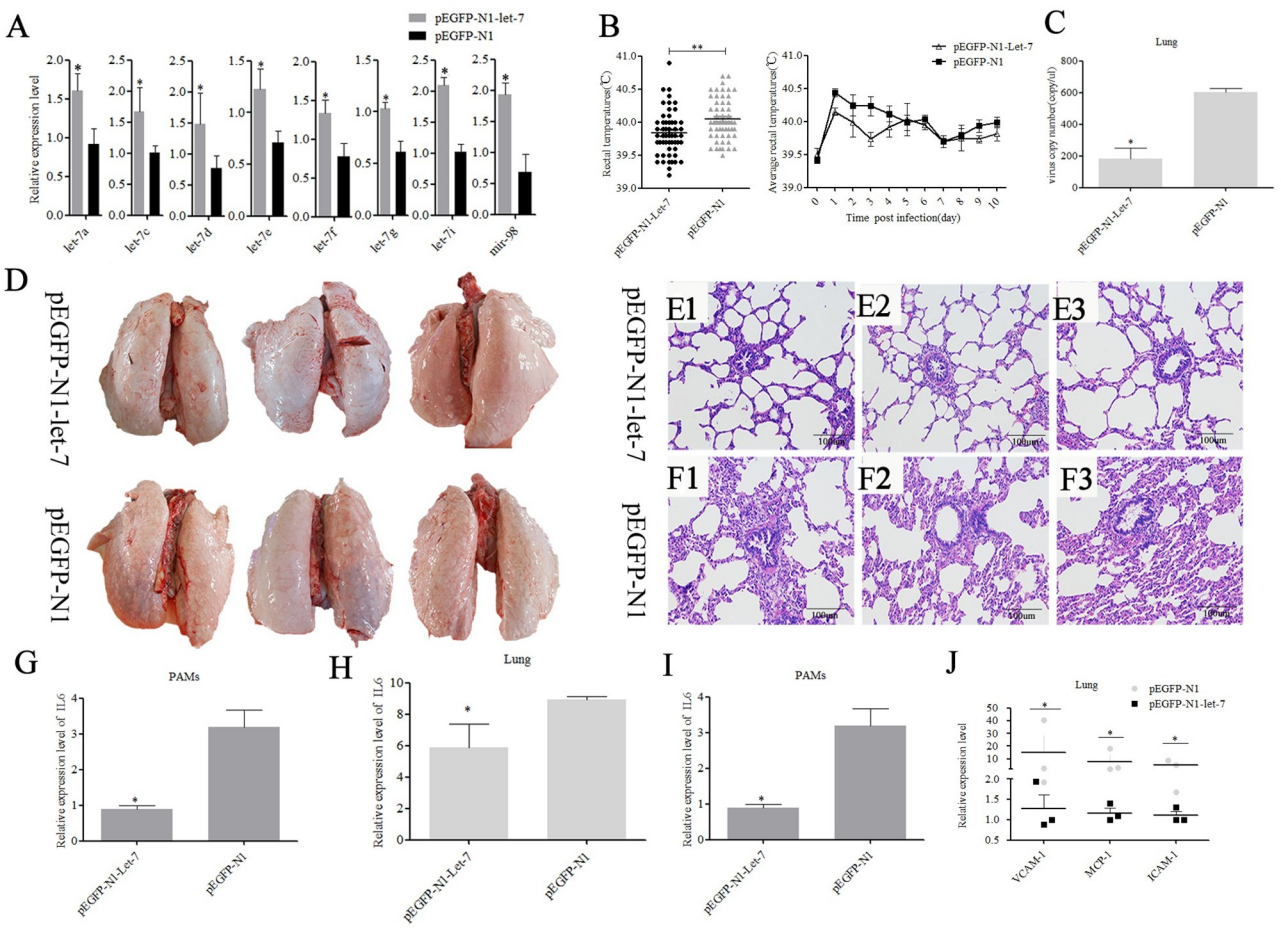
pEGFP-N1-Let-7 exhibits antiviral activity by intramuscular injection *in vivo*. The expression of let-7 family was detected in lung of pigs from the two groups (A). Rectal temperature of pigs from two groups was compared after PRRSV-2 infection (B). The viral loads in the lungs of the two groups were detected by absolute quantitative PCR (C). The pathological changes of lung surface were observed. After lung removal, the lung surface was rinsed with PBS, and then the lungs were placed on a white background plate and photographed. All six lungs are photographed at once (D). Histopathological analyses were performed on the lungs of pigs from both groups (E, F). The viral loads in the PAMs of pigs from the two groups were detected by absolute quantitative PCR (G). IL6 expression was detected in lung (H) and PAMs of pigs from the two groups (I). The expression of MCP-1, VCAM-1, and ICAM-1 was detected in lung of pigs from the two groups (J). Data are expressed as the mean± standard deviation of three independent experiments. P values were calculated using a non-parametric Mann-Whitney test. *p< 0.*05* and**p< 0.*01*.

Four-week-old piglets were randomly divided into two groups (n = 3 in each group). The piglets were infected with 10^5.7^ TCID50 of the PRRSV-2 strain WuH3 at 5 h after injection (2.5 mg/kg) of plasmid pEGFP-N1-let-7 (the experimental group) or pEGFP-N1 (the NC group). The RT–qPCR results showed that the expression of the all eight let-7 family members in the pEGFP-N1-let-7 group was significantly higher than that in the pEGFP-N1 group (Fig 7A). The experimental group piglets exhibited a lower rectal temperature than the NC group piglets (Fig 7B). The NC group displayed a range of clinical signs, including inappetence, lethargy, dyspnea, eyelid edema and purple surface on the abdomen. On the tenth day post-infection, all the piglets were sacrificed, and lungs and PAMs were collected. Severe interstitial pneumonia and pulmonary hemorrhages were observed in NC group piglets (Fig 7D). The NC group piglets also presented obvious microscopic lung lesions with more severe lesions, including a disappeared lung structure, numerous inflammatory cells and necrotic debris infiltrated in alveolar spaces and bronchioles, compared with the experimental group piglets (Fig 7E and 7F). The copy number of PRRSV-2 was significantly lower in the lungs and PAMs (*p<0*.*05*, decreased by more than 90%) of the experimental group piglets compared with that of the NC group (Fig 7G). IL6 expression was also significantly reduced in the lungs and PAMs (*p<0*.*05*) of the experimental group (Fig 7H and 7I). In addition, pEGFP-N1-let-7 also significantly (*p<0*.*05*) reduced the expression of *MCP-1*, *VCAM-1*, and intercellular adhesion molecule 1 (*ICAM-1*) in the lungs of the experimental group piglets (Fig 7J). This means that the degree of inflammation/injury in the lungs of the control group is more severe than that of the pEGFP-N1-let-7 experimental group.

## Discussion

Previous studies indicated that the susceptibility of different pig breeds to PRRSV was different. In this study, it was confirmed that the PRRSV-2 copy number was different in PAMs from 4 pig breeds during 24–72 h post-infection. The viral loads of PAMs in Qingping and Meishan pigs were lower than those in Pietrain and Landrace pigs. The expression of the let-7 family in PAMs from Pietrain pigs was significantly lower than that in PAMs from Meishan, Landrace and Qingping pigs. In addition, the let-7 family might inhibit PRRSV-2 replication by binding to the PRRSV-2 3’UTR. This may be one of the reasons why Pietrain pigs’ resistance to PRRSV-2 is significantly lower than that of the other three pig breeds. In the early stages (9 hpi) of PRRSV-2 infection, the expression level of the let-7 family was decreased in Meishan pigs and increased in Pietrain pigs after PRRSV-2 infection. Furthermore, the expression levels of *NEAT1* and ARID3A, which inhibit the let-7 family, were significantly increased in PAMs from Meishan pigs and reduced in PAMs from Pietrain pigs. Previous studies indicated that the first 8–10 h of initial infection is critical for the virus to divert host signaling and set up an intracellular environment more beneficial to viral growth [24]. The PRRSV-2 infection promoted the expression of *NEAT1* and *ARID3A* and inhibited the expression of let-7, which created excellent conditions for PRRSV-2 replication in PAMs from Meishan pigs. It may be that it is not necessary for Pietrain pigs to establish a beneficial environment for viral growth due to their low resistance to PRRSV-2. The increase in the let-7 family expression in PAMs from Pietrain pigs inhibited PRRSV-2 replication. Overall, the *NEAT1*/*ARID3A*/let-7 family may have important contributions to the susceptibility of different pig breeds to PRRSV-2.

*IL-6* is a multifunctional cytokine that is produced by a wide range of cells and plays a critical role in the progression of lung inflammation/injury [25]. Previous studies have shown that PRRSV infection causes an increase in *IL6* expression levels and leads to lung lesions [26,27]. Our results also confirmed that *IL6* could promote the expression of NF-κB, a central transcription factor and a pleiotropic regulator of many genes involved in acute lung injury [28], resulting in sequential signaling cascades and upregulating the expression of proinflammatory cytokines such as *MCP-1*, *VCAM-1*, and *ICAM-1*, which was consistent with previous studies [29–31]. Lung inflammation/injury, which is associated with systemic inflammatory responses, is a common problem with significant morbidity and mortality [32]. A series of adhesion molecules (e.g., *VCAM-1* and *ICAM-1*) and cytokines (e.g., *MCP-1*, *IL-6*, and NF-κB) are expressed in inflammatory disorders. Therefore, medications that suppress the expression of proinflammatory cytokines are promising candidates for the treatment and prevention of chronic inflammatory diseases [33]. Previous studies have shown that *IL-6* plays an important role in both primary and secondary storms; thus, inhibition of *IL-6* has potential value in the treatment of lung inflammation/injury [34]. This is consistent with the constructed plasmid pEGFP-N1-let-7 reducing *IL6*, NF-κB, *MCP-1*, *VCAM-1*, and *ICAM-1* expression by approximately 10 times and attenuating lung injury induced by PRRSV *in vivo*. In addition, with PRRSV infection, *IL6* was differentially expressed among different pig breeds, which is consistent with previous reports [24,35] (S3 Fig). Our results confirmed that the let-7 family could significantly inhibit the expression of *IL6*. The let-7 family is also differentially expressed among 4 pig breeds and has an opposite trend with *IL6*, suggesting that let-7/*IL6* may play an important role in the susceptibility of different breeds of pigs to PRRSV-2. On the other hand, previous studies have revealed that PRRSV-2 infection activates NF-κB signaling in Marc-145 cells and PAMs by inducing IκB degradation and p65 nuclear translocation. Additionally, *NF-κB* was required for optimal PRRSV replication. [36,37] Thus, the let-7 family inhibited NF-κB activity through *IL6*, thereby inhibiting PRRSV-2 replication.

*NEAT1* is known as virus-inducible noncoding RNA [38] and binds with many miRNAs, acting as a miRNA sponge, thus removing the inhibitory effect of miRNA on target genes [39,40]. Our results shown that the expression of *NEAT1* and let-7 had a significantly negative correlation in PAMs and found that *NEAT1* could regulate let-7 through all three binding sites (S1 Fig). Meanwhile, let-7e overexpression reduced the expression of *NEAT1*, while let-7e inhibition increased *NEAT1* expression. This result suggested that *NEAT1* and let-7e participate in a reciprocal repression feedback loop. PRRSV-2 could induce the expression of *NEAT1*, inhibiting the let-7 family, thereby reducing the anti-PRRSV effect of let-7 and setting up a beneficial environment for PRRSV replication. The overexpression of let-7 could also inhibit the replication of PRRSV-2 by inhibiting *NEAT1*.

MiRNAs are first transcribed in the nucleus [41]. Therefore, ARID3A could affect the formation of pre-let-7 by binding to the promoter of let-7a-2/let-7f-2/let-7d in the nucleus. In addition, Western blot results showed that the expression of ARID3A was upregulated after PRRSV-2 infection. Together, PRRSV-2 could also evade the host’s immune response by increasing *ARID3A* expression to inhibit let-7 transcription. This phenomenon reveals how PRRSV infection regulates abnormal miRNA expression in host cells.

**Fig 8:**
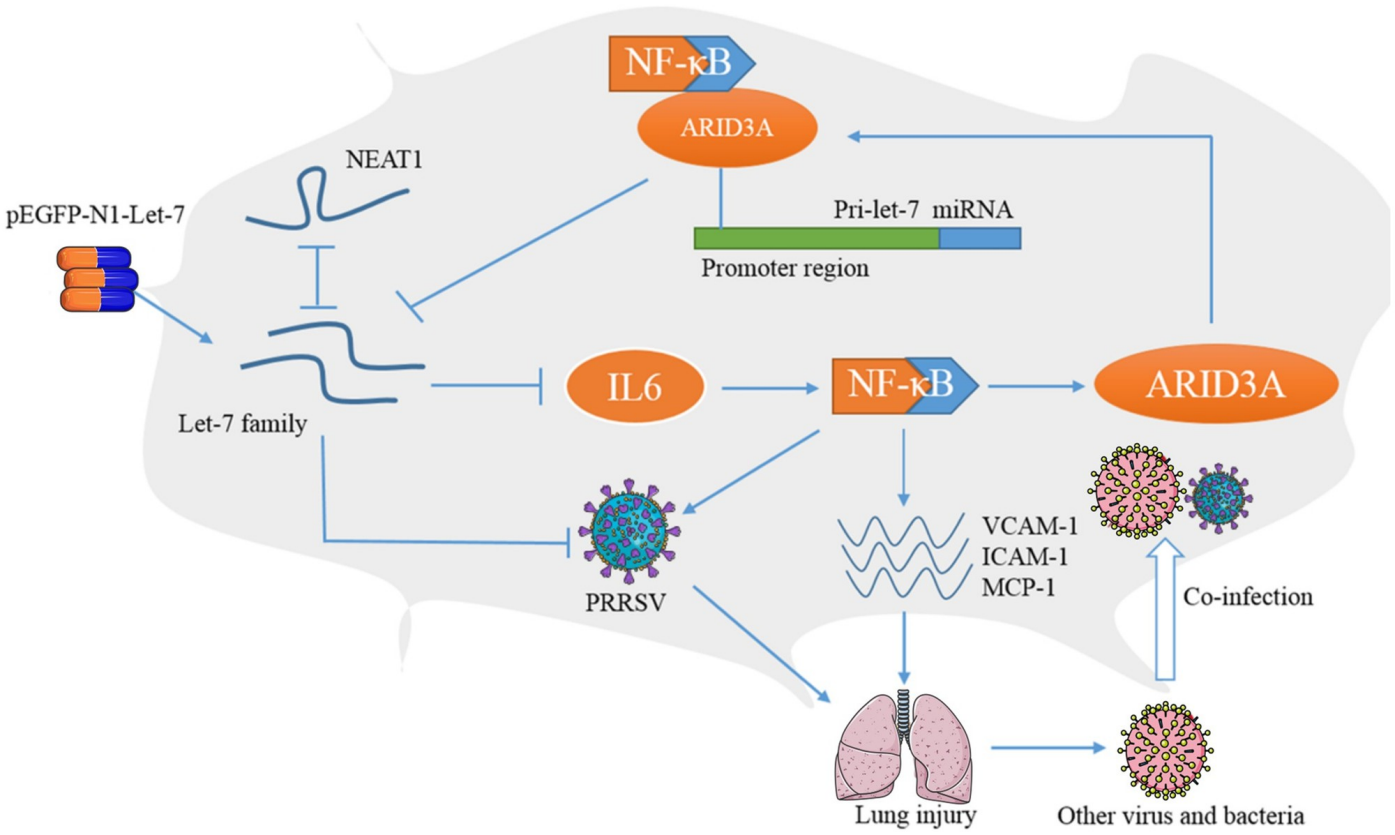
Diagram of NEAT1/ARID3A/NF-κB/let-7/IL6 regulation during PRRSV-2 infection (drawn using PowerPoint). **P**arts of Fig 8 were drawn by using pictures from Servier Medical Art, provided by Servier, licensed under a Creative Commons Attribution License.

In summary, the let-7 family is regulated by NEAT1 and ARID3A/NF-κB, which are necessary for PRRSV replication. Moreover, pEGFP-N1-let-7, which coexpressed let-7 family members, significantly reduced viral infection and pathological changes in PRRSV-infected piglets (Fig 8).

## Materials and methods

### Ethics statement

All animal procedures were approved (HZAUSW2015-018) by the Scientific Ethics Committee of Huazhong Agricultural University, Wuhan, China.

### Cells and virus

PAMs were isolated by lung lavage from 5-week-old Meishan (MS), Landrace (L), Pietrain (P) and Qingping (QP) pigs as described [42,43]. All pigs tested negative for anti-PRRSV specific antibody and PRRSV antigen by ELISA and RT–PCR assays. Marc-145 cells, a monkey kidney cell line highly permissive for PRRSV infection, were obtained from the State Key Laboratory of Agricultural Microbiology (Huazhong Agricultural University, Wuhan, China). PAMs and Marc-145 cells were cultured in RPMI 1640 medium (Invitrogen, Carlsbad, CA, USA) supplemented with 10% fetal bovine serum (Gibco, Grand Island, NY, USA), 100 U/mL penicillin and 100 mg/mL streptomycin in a humidified 37°C/5% CO_2_ incubator. The PRRSV-2 strain WuH3 (GenBank accession NO. HM853673) was used to infect PAMs and Marc-145 cells. The virus was also taken from the State Key Laboratory of Agricultural Microbiology, Huazhong Agricultural University, and Wuhan, China. Virus titers were determined via immunofluorescence staining of the PRRSV nucleocapsid protein in Marc-145 cells and calculated by the Reed-Muench method [44]. The titer of this pool was 10^5.7^ TCID50.

### Sample preparation

*In vitro* experimental infection with the PRRSV-2 strain WuH3 was performed on PAMs isolated by bronchoalveolar lavages from MS, L, P and QP pig breeds, with a multiplicity of infection (MOI) of 0.1 PFU/cell. The PRRSV-infected PAMs were collected at 9 hpi, 36 hpi and 60 hpi, and the PAMs of 5 pigs of each breed were evenly mixed. The control group PAMs were mock-infected with culture medium and collected at 9 h, 36 h, and 60 h. Total cellular RNA was prepared using the TRIzol reagent (Invitrogen, Cashman, CA, USA) according to the manufacturer’s instructions.

### MiRNA library construction and deep sequencing

All RNA samples were quantified and examined for protein contamination (A260 nm/A280 nm ratios) and reagent contamination (A260 nm/A230 nm ratios) by a Nanodrop ND 2000 spectrophotometer (Thermo Scientific, MA, USA). Twenty-four miRNA libraries were constructed. Each library was made from pooled equimolar amounts of total RNA from PAMs of 4 pig breeds at 3 time points. Total RNA was prepared for small RNA sequencing by synthesis according to the procedure and standards of the Illumina Sample Preparation Protocol. Deep sequencing was performed by the Illumina/Solexa Genome Analyzer (BGI, Shenzhen, China).

### Sequencing data analysis

The raw sequence reads were filtered for composition, presence of adaptor dimers, length, sequence repetition, and copy numbers using SOAP V2.0 [45]. To determine conserved miRNAs, the filtered sequences were initially used to search miRBase with BLASTN, which allowed a maximum of two mismatches, and the gaps were counted as mismatches. The criterion was then implemented according to the reported miRNA protocol [46]. Comparison of the known miRNA expression between the two libraries was conducted to determine the DE-miRs. MiRNAs were first filtered to retain only those with a mean expression value of at least 28 read counts between the two groups [17]. The expression of miRNA was normalized and shown in two libraries by calculating fold-change and p-value [18,19]. A miRNA was labeled as differentially expressed when |log2 (fold change)| ≥1 and p-value ≤0.01. These DE-miRs were further verified by RT–qPCR analysis.

### Prediction of miRNA target genes and conservation analysis

ViTa was used to predict miRNA binding sites in the PRRSV genome. For the conservative analysis of PRRSV, we aligned the potential target sequences in several representative PRRSV strains collected from GenBank. Potential target genes for the let-7 family were predicted by MiRanda, Target Scan, PicTarand, and RNA hybrid 2.2.

### Dual luciferase reporter assay

For each potential target gene of the let-7 family, miRNA binding sites were amplified from pig genomic DNA using gene-specific primers. The primer sequences are available in S1 Table. Using the *Sac* I and *Xho*I restriction sites within the vector, each PCR product was cloned into the pmirGLO vector (Promega, Madison, Wisconsin, USA) and named IL6 3’UTR-WT, PRRSV3’UTR-WT, NEAT1-WT1, NEAT1-WT2, and NEAT1-WT3. The theoretical let-7 family binding sequences in *IL6*, PRRSV, and NEAT1 were mutated as indicated (IL6 3’UTR-MUT, PRRSV3’UTR-MUT, and NEAT1-MUT3). Marc-145 cells were transfected with a mixture of luciferase reporter plasmids, miRNA mimics or miRNA inhibitors using Lipofectamine 2000 (Invitrogen, Carlsbad, CA, USA) according to the manufacturer’s instructions. The sequences of let-7 family mimics/inhibitors are listed in S2 Table. Cells were maintained in a 24-well plate in DMEM with 10% FBS, penicillin (100 U/ml), and streptomycin (100 mg/ml) at 37°C with 5% CO_2_. At 24 h posttransfection, cells were lysed in passive lysis buffer (Promega, Madison, Wisconsin, USA), and firefly and Renilla luciferase activities were then measured using the Dual Luciferase Reporter Assay System (Promega, Madison, Wisconsin, USA) according to the manufacturer’s instructions.

### RNA isolation and RT–qPCR

Total RNA from Marc-145 cells, PAMs and supernatant PRRSV were extracted with TRIzol (Invitrogen, Carlsbad, CA, USA) following the manufacturer’s instructions, and then M-MLV Reverse Transcriptase was used for reverse transcription according to the manufacturer’s protocol (Promega, Madison, USA). RT–qPCR analysis was performed using a LightCycler 480 Real-Time PCR Detection System (Roche, Basel, Switzerland) and SYBR Green Real-Time PCR MasterMix (Toyobo, Osaka, Japan). The relative expression level was analyzed using the 2^-ΔΔCT^ method. The PRRSV copy number within each sample was calculated using absolute (standard curve) quantification. Gene-specific forward and reverse primers are listed in S3 Table.

### Western blot

Marc-145 cells and PAMs were collected and lysed using RIPA lysis buffer supplemented with fresh protease and phosphatase inhibitors. The protein concentration was determined by the BCA Protein Assay kit (Solarbio, Beijing, China). Equal amounts of each sample were loaded and subjected to SDS–PAGE, transferred onto PVDF membranes (Millipore, Darmstadt, Germany), and then incubated for 12 h at 4°C with the following primary antibodies: IL6 (sc-323975, Santa Cruz, USA), PRRSV nucleocapsid protein (GTX129270, GeneTex, Alton Pkwy Irvine, CA, USA), NF-κB p65 (A11201, Abclonal, Wuhan, China), ARID3A (A7668, Abclonal, Wuhan, China), β-actin (AC026, Abclonal, Wuhan, China), anti-β-tubulin (GB11017B, Servicebio, Wuhan, China), and H3 (A2348, Abclonal, Wuhan, China). After incubation, the PVDF membrane was washed with Tris-buffered saline Tween 20 (TBST) three times and then incubated with HRP-conjugated secondary antibodies (G1213, Servicebio, Wuhan, China). The protein on this membrane was visualized using enhanced chemiluminescence (ECL) (Servicebio, Wuhan, China) in a Western Blotting Detection System.

### Plasmids construction

The pCDNA3.1-IL6 and pCDNA3.1-ARID3A eukaryotic expression plasmids were constructed. Using cDNA as a template, the coding sequences of the *IL6* and *ARID3A* genes were amplified with gene-specific primers. The PCR products were digested with *Hin*d III and *Eco*R I (Thermo Scientific, MA, USA) and cloned into the pCDNA3.1 vector (Promega, Madison, USA) using T4 DNA Ligase (Takara, Japan). The let-7 family expression plasmid pEGFP-N1-let-7 was constructed in accordance with previous studies. [47,48] The precursor and flanking sequences of the let-7 family were obtained from NCBI. Then, the sequences of pre-let-7a-1, pre-let-7a-2, pre-let-7c, pre-let-7d, pre-let-7e, pre-let-7f-1, pre-let-7f-2, pre-let-7g, pre-let-7i, and pre-mir-98 along with their flanking sequences were amplified from genomic DNA by PCR using specific primers (containing sequences of 20 bp each of adjacent miRNA), and fusion PCR was used to connect pre-let-7a-1, pre-let-7a-2, pre-let-7c, pre-let-7d, pre-let-7e, pre-let-7f-1, pre-let-7f-2, pre-let-7g, pre-let-7i, and pre-mir-98 into miRNA clusters of 1686 bp. The purified PCR product was digested with relevant restriction enzymes (*Big*I II and *Pst* I) and inserted into the pEGFP-N1 vector. A stop codon was added between the EGFP gene and multiple cloning sites to stop the translation of EGFP. Nine let-7a-2/let-7f-2/let-7d promoter deletion fragments (D1-D9) were amplified with specific primers. PCR products were digested with *Sac*I and *Xho*I (Thermo Scientific, MA, USA) and then subcloned into the luciferase reporter vector pGL3-Basic (Promega, Madison, Wisconsin, USA). The ARID3A binding sequences in let-7a-2/let-7f-2/let-7d were mutated as indicated. The primer sequences for plasmid construction are listed in S1 Table. The pCDNA3.1-NEAT1 plasmid was stored in our laboratory.

### Electrophoretic mobility shift assay (EMSA)

Nuclear extracts were prepared from PAMs with the Nuclear Extraction Kit (Beyotime, Jiangsu, China). EMSA was performed as described previously. Oligos corresponding to the ARID3A-binding sites of the let-7a-2/let-7f-2/let-7d core promoter were synthesized and annealed into double strands. The DNA-binding activity of the ARID3A protein was detected by a Light-Shift Chemiluminescent EMSA Kit (Thermo Fisher Scientific, Waltham, MA, USA). The probe sequences for EMSA are listed in S4 Table.

### Chromatin immunoprecipitation (ChIP) assay

The ChIP experiments were conducted to assess the binding of endogenous ARID3A to the let-7a-2/let-7f-2/let-7d promoter in PAMs using a ChIP Assay Kit (Beyotime, Jiangsu, China). Precleared chromatin was incubated with the ARID3A antibody (Abclonal, Wuhan, China) or the nonimmune IgG (Beyotime, Jiangsu, China) control overnight at 4°C. The immunocomplexes were isolated on Protein A agarose beads with a ChIP Assay Kit (Beyotime, Jiangsu, China). The chromatin complexes were eluted from the beads, and DNA cross-linking was subsequently reversed. Purified DNA from the samples and the input controls was analyzed for the presence of let-7a-2/let-7f-2/let-7d promoter sequences containing putative ARID3A response elements using PCR, and the primers are listed in S5 Table.

### Immunoprecipitation

Marc-145 cells cultured in 10-cm-diameter dishes were collected and lysed in nondenaturing lysis buffer (Sangon, Shanghai, China) supplemented with protease and phosphatase inhibitor cocktails. An equal mass of lysate was incubated overnight with 2 μg of anti-ARID3A (A7668, Abclonal, Wuhan, China), anti-p65 (A11201, Abclonal, Wuhan, China) or anti-IgG (Beyotime, Jiangsu, China) together with 25 μl of Protein A+G Agarose beads (Beyotime, Jiangsu, China). After centrifugation, the beads were washed with 1 mL of lysate buffer three or four times. Then, 15 μl of 2×SDS loading buffer was added to the beads, boiled for 10 min, and loaded onto an SDS–PAGE gel for western blot analysis.

### Immunofluorescence labeling assay

Marc-145 cells were immediately fixed with 4% paraformaldehyde for 30 min. For immune detection, the cells were permeabilized in 0.1% Triton X-100 (Sigma–Aldrich, St. Louis, MO) for 20 min at room temperature and incubated with 10% normal goat serum for 1 h. After blocking nonspecific binding, the coverslips were incubated overnight with mouse monoclonal anti-ARID3A antibody (sc-398367, Santa Cruz, CA, USA) and anti-PRRSV N protein (GTX129270, GeneTex Inc., Irvine, CA, USA) at 4°C. The cells were then incubated with the corresponding secondary antibody (Servicebio, Wuhan, China) in PBS for 2 h at room temperature. The coverslips were then stained with DAPI (Servicebio, Wuhan, China). The stained cells were subsequently observed using an Olympus BX-51 fluorescence microscope (Olympus, Tokyo, Japan).

### Immunohistochemistry

ARID3A immunohistochemical staining was evaluated in fixed lungs of uninfected pigs and PRRSV-infected pigs. Inflation-fixed lungs were washed in phosphate-buffered saline (PBS) three times and separated for paraffin embedding. The paraffin-embedded lungs were sectioned at 6 μm for immunohistochemical staining of ARID3A, and tissue sections were deparaffinized by washing in xylene three times for 10 min each, followed by rehydration through a series of ethanol washes from 100% to 70% ethanol. Slides were placed in methanol containing 0.5% hydrogen peroxide for the removal of endogenous peroxidase activity. Nonspecific binding was blocked by incubation of the slides for 1 h at room temperature in 5% BSA. Lung sections were incubated with anti-ARID3A antibody (A7668, ABclonal, Wuhan, China) overnight at 4°C. Sections were rinsed in 0.1 M PBS (pH 7.2–7.4) five times for 5 min each and incubated for 30 min at room temperature with secondary antibody. Sections were washed with PBS five times for 5 min each, incubated with diaminobenzidine for 6 min, washed with running water for 6 min, stained with hematoxylin for 40 s and washed with running water for 5 min. In addition, the primary antibody was substituted with PBS, and the controls were performed under the same conditions described earlier.

### Animal experiment

Six four-week-old piglets were divided into two groups: the pEGFP-N1-let-7 treatment group and the pEGFP-N1 control group. PEGFP-N1-let-7 or pEGFP-N1 (2.5 mg/kg of body weight per dose) was mixed with D5W solution to finally obtain a 3-ml mixture solution. This 3-mL solution was administered to piglets through intramuscular injection. At 5 h post-intramuscular injection, 1.5 mL of the PRRSV-2 strain WuH3 (10^5.2^ TCID50) was administered to piglets. The rectal temperature was measured twice a day. On day 10, we performed pathological dissection and collected all the lungs and PAMs of the piglets. The sacrificed pigs were removed, and the animal experiments were performed by random and blinded methods. In the *in vivo* experiment, there were three replicates for each of the two groups. All experiments were performed at least three times in triplicate, excluding average rectal temperatures.

### Statistical analysis

All experiments were performed at least three times. Data are presented as the mean ± SD. Statistical analyses between different groups were performed using the t-test. The nonparametric Mann–Whitney statistical test was used in the *in vivo* experiment due to the small number of animals available. A p value of less than 0.05 was considered statistically significant, and a p value of less than 0.01 was considered highly statistically significant.

## Supporting information

S1 Fig Let-7 interacts with NEAT1. Dual-luciferase reporter assay was performed in Marc-145 cells co-transfected with NEAT1-MUT3 and let-7e mimics/inhibitor (A). Dual-luciferase reporter assay was performed in Marc-145 cells co-transfected with NEAT1-WT1/NEAT1-WT2/NEAT1-WT3 and pEGFP-N1-Let-7 (B). Data are from three independent experiments (mean± SD). *p< 0.*05* and**p< 0.*01*.
(TIF)

S2 Fig pEGFP-N1-let-7 significantly inhibits PRRSV-2 replication and IL6 expression in PAMs. The let-7 family co-expression plasmid pEGFP-N1-let-7 was constructed by fusion PCR (A). PAMs transfected with pEGFP-N1-let-7 or pEGFP-N1 (4μg), cells were harvested at the indicated times post infection, and the copy number of infectious virus was quantified by absolute quantitative PCR (B) and PRRSV-2 N protein was analyzed by Western blot (C). Immunofluorescence assay revealed PRRSV-2 N protein expression in Marc-145 cells transfected with pEGFP-N1-let-7 and infected with PRRSV-2 for 9 h, 36 h (D). The mRNA and protein expression level of IL6 were also detected by RT-qPCR (E) and Western blot (F). Data are from three independent experiments (mean± SD), *p< 0.*05* and**p< 0.*01*.
(TIF)

S3 Fig The mRNA expression of *IL6* was quantitatively detected in PAMs from different pig breeds at different time after PRRSV-2 infection by RT-qPCR. (TIF)

S1 Table Sequences of primers used for plasmid construction in this study. (DOCX)

S2 Table Sequences of microRNA (miRNA) mimics/inhibitors and siRNA used in this study. (DOCX)

S3 Table Sequences of primers used for RT-qPCR in this study. (DOCX)

S4 Table Probe sequences for EMSA. (DOCX)

S5 Table PCR primer sequences for ChIP assay. (DOCX)
