# Supplementary figures and images for: miRNA let-7 family regulated by NEAT1 and ARID3A/NF-κB inhibits PRRSV-2 replication in vitro and in vivo
Source: PLoS Pathog. 2022 Oct 10;18(10):e1010820. doi: 10.1371/journal.ppat.1010820 (PMC9550049; doi:10.1371/journal.ppat.1010820)

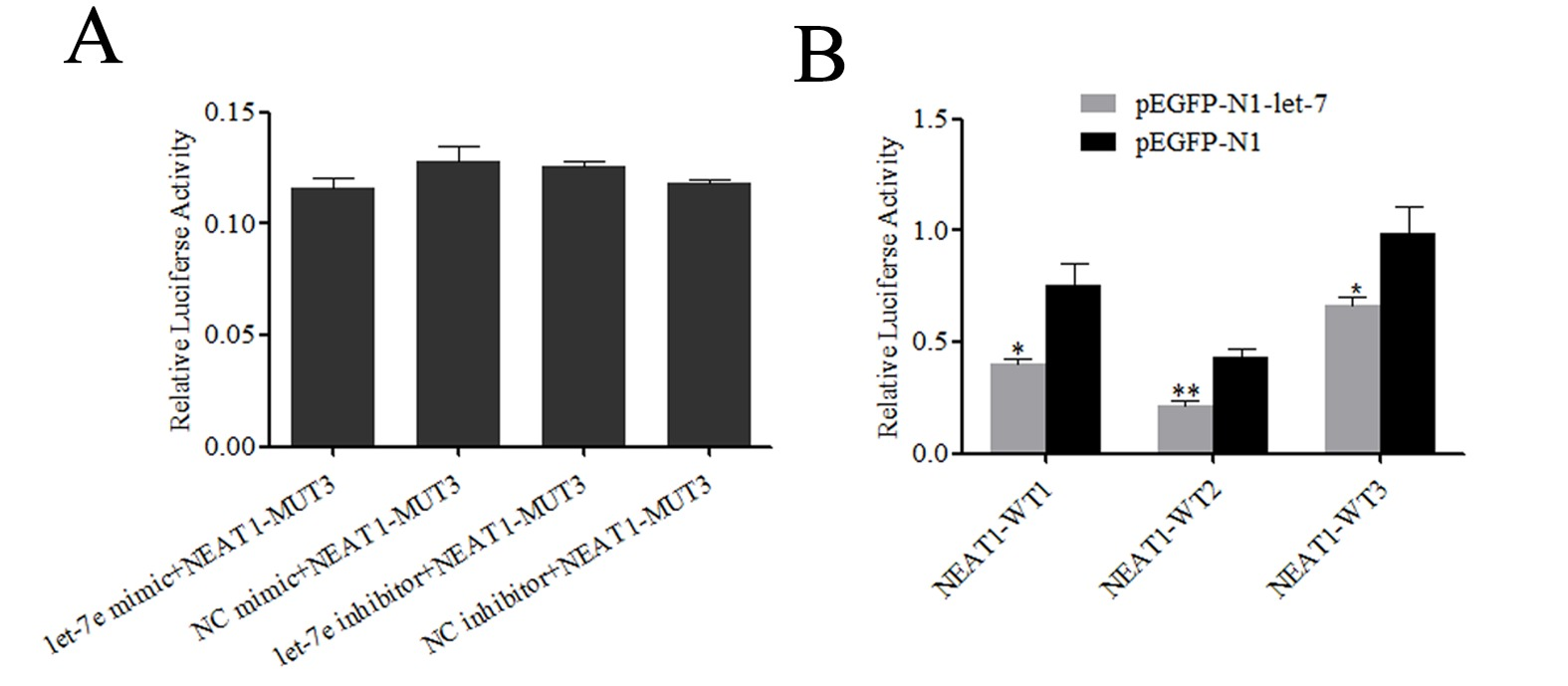

Supplement: S1 Fig — Dual-luciferase reporter assay was performed in Marc-145 cells co-transfected with NEAT1-MUT3 and let-7e mimics/inhibitor (A). Dual-luciferase reporter assay was performed in Marc-145 cells co-transfected with NEAT1-WT1/NEAT1-WT2/NEAT1-WT3 and pEGFP-N1-Let-7 (B). Data are from three independent experiments (mean± SD). *p< 0.05 and**p< 0.01. (TIF) [file ppat.1010820.s001.tif]

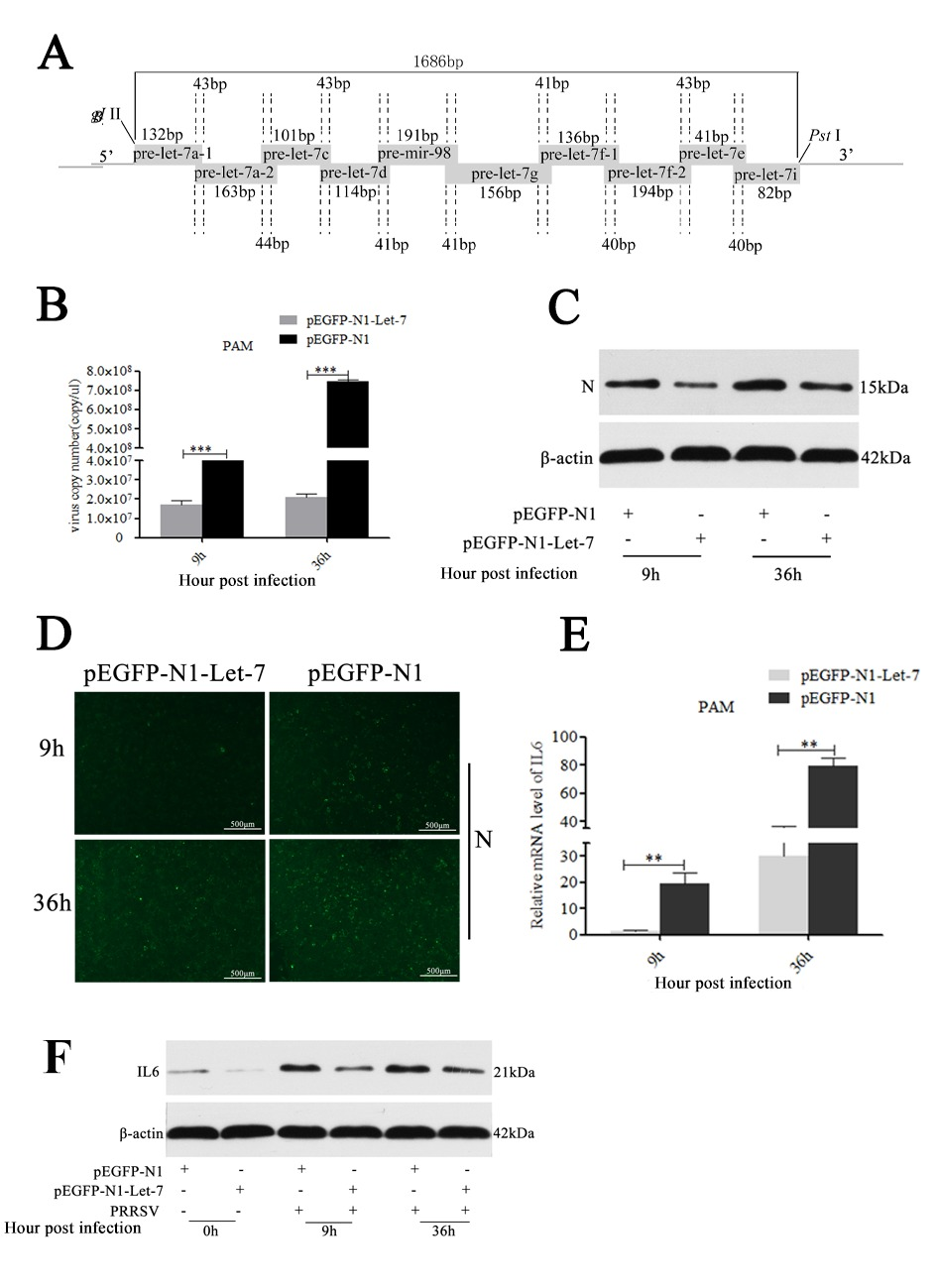

Supplement: S2 Fig — The let-7 family co-expression plasmid pEGFP-N1-let-7 was constructed by fusion PCR (A). PAMs transfected with pEGFP-N1-let-7 or pEGFP-N1 (4μg), cells were harvested at the indicated times post infection, and the copy number of infectious virus was quantified by absolute quantitative PCR (B) and PRRSV-2 N protein was analyzed by Western blot (C). Immunofluorescence assay revealed PRRSV-2 N protein expression in Marc-145 cells transfected with pEGFP-N1-let-7 and infected with PRRSV-2 for 9 h, 36 h (D). The mRNA and protein expression level of IL6 were also detected by RT-qPCR (E) and Western blot (F). Data are from three independent experiments (mean± SD), *p< 0.05 and**p< 0.01. (TIF) [file ppat.1010820.s002.tif]

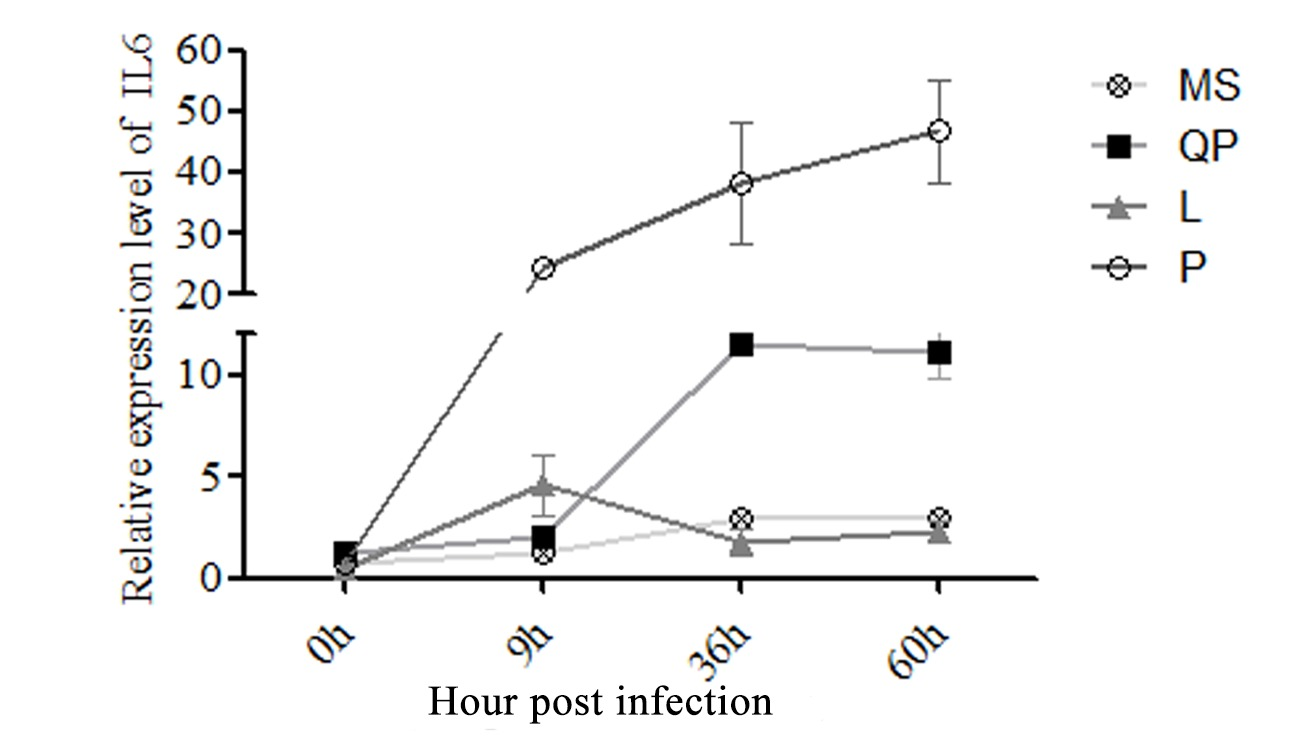

Supplement: S3 Fig — (TIF) [file ppat.1010820.s003.tif]
