# Supplementary material for: miRNA let-7 family regulated by NEAT1 and ARID3A/NF-κB inhibits PRRSV-2 replication in vitro and in vivo
Source: PLoS Pathog. 2022 Oct 10;18(10):e1010820. doi: 10.1371/journal.ppat.1010820 (PMC9550049; doi:10.1371/journal.ppat.1010820)
Supplement: S1 Table — (DOCX) [file ppat.1010820.s004.docx]

**Table S1 sequences of primer used for plasmid construction in this study.**

| Primer | Sequence (5’-3’) |
| --- | --- |
| PRRSV-F-WT | CGAGCTCTGGCATTCTTTGGCACCTCAG |
| PRRSV-R-WT | CCGCTCGAGAATTACGGCCGCATGGTTCTC |
| IL6-F-WT | CGAGCTCGGTATGAGCGTTAGGACAC |
| IL6-R-WT | CCGCTCGAGCGGCAGGGCGTTTATTGGTAT |
| Ssc-let-7a1-F | GAAGATCTTCTAATTCTCAGCATGTTGCTTTGTGC |
| Ssc-let-7a1-R | GGTAATCCTGGTTCTCCTGTGTAAGAAATGGTAGCTTGCCCGC |
| Ssc-let-7a2-F | GCGGGCAAGCTACCATTTCTTACACAGGAGAACCAGGATTACC |
| Ssc-let-7a2-R | CCACTGTTGCTTCAAACTCCTTGATGCTGTCTGGATGCAGATTT |
| Ssc-let-7c-F | AAATCTGCATCCAGACAGCATCAAGGAGTTTGAAGCAACAGTGG |
| Ssc-let-7c-R | TCTTGCCAGTCTTCCACTTAACGATGAAGAATTCCTCGATGGC |
| Ssc-let-7d-F | GCCATCGAGGAATTCTTCATCGTTAAGTGGAAGACTGGCAAGA |
| Ssc-let-7d-R | AGGTGGAAGGGGACAGGTAACAAGGAAACTGGTTACCGTGG |
| Ssc-let-7e-F | AGACTTGCTGCATTTTGTTTGGCTGAGGTAGGAGGTTG |
| Ssc-let-7e-R | CAAACTACTACCTCAGCCAGCTGGGGAAAGCTAGGAGGCC |
| Ssc-let-7f1-F | GGGAAACCAGGTAATGGGGAGAGAACACAGCACAGCATAAT |
| Ssc-let-7f1-R | TATCCCCACCCAACCTGTATGTGGGCTGAAGATGAACACT |
| Ssc-let-7f2-F | AGTGTTCATCTTCAGCCCACATACAGGTTGGGTGGGGATA |
| Ssc-let-7f2-R | GGCTGAGGTAGGAGGTTGAAACAAAATGCAGCAAGTCT |
| Ssc-let-7g-F | CTCTAGAAGGGCAGGAGGCATCCTCCAACGCTCCGTATCCT |
| Ssc-let-7g-R | ATTATGCTGTGCTGTGTTCTCTCCCCATTACCTGGTTTCCC |
| Ssc-mir-98-F | CCACGGTAACCAGTTTCCTTGTTACCTGTCCCCTTCCACCT |
| Ssc-mir-98-R | AGGATACGGAGCGTTGGAGGATGCCTCCTGCCCTTCTAGAG |
| Ssc-let-7i-F | GGCCTCCTAGCTTTCCCCAGCTGGCTGAGGTAGTAGTTTG |
| Ssc-let-7i-R | CTGCAGAACCAATGCATCTAGCAAGGCAGTAGCTTGCGC |
| pCDNA-IL6-F | CAAGCTTGAGCCCACCAGGAACGAAAGA |
| pCDNA-IL6-F | GGAATTCCTGTGCCCAGTGGACAGGTTT |
| Let-7a2/7f2-F | CGAGCTCGGCTGGTTTTCCTCTTGGTTCT |
| Let-7a2/7f2-R | CCCTCGAGGGCTGATGTTGAGTGATGCTTGG |
| D1-F | CGAGCTCGAATTGGCTGAGGTATGGTGAG |
| D2-F | CGAGCTCGCAGAGTGTCAGTGAGTGAGCAG |
| D3-F | CGAGCTCGTTTTGATAAACTGCTAAACTTTTCC |
| D4-F | CGAGCTCGTGATGACTAATAAATGATGTGAGGTT |
| D5-F | CGAGCTCGATGGGGTTTATTTCTGGATGT |
| D6-F | CGAGCTCGAATCTTTACAGTGGCTGCTTC |
| D7-F | CGAGCTCGAAACTAAACTGGTCATATTGCTT |
| D8-F | CGAGCTCGCAGGCTAAGGGTCTAATCGGA |
| D9-F | CGAGCTCGTAGATTATGTTAAGTGAATGAAAGGAG |
| P-ARID3A-F | GGAATTCC ATGAAGCTGCAGGCTGTGATGGAGA |
| P-ARID3A-R | CAAGCTTGTTAAGGCAATGAGTTATTTGAGGTAG |
| NEAT1-F-WT1 | CGAGCTCGGTGGCAGAGTCAGGCGATAC |
| NEAT1-R-WT1 | CCTCGAGGGACAAAGCAGGGCAAAGG |
| NEAT1-F-WT2 | CGAGCTCGCTGTTGGAGTCGGTACTGC |
| NEAT1-R-WT2 | CCTCGAGGCTGCTTGGGACTTGGAAC |
| NEAT1-F-WT3 | CGAGCTCGAAACTTGAGCAGAGTGAAAA |
| NEAT1-R-WT3 | CCTCGAGGCAAACTAAAGATACAGCGGAT |
