# Supplementary material for: miRNA let-7 family regulated by NEAT1 and ARID3A/NF-κB inhibits PRRSV-2 replication in vitro and in vivo
Source: PLoS Pathog. 2022 Oct 10;18(10):e1010820. doi: 10.1371/journal.ppat.1010820 (PMC9550049; doi:10.1371/journal.ppat.1010820)
Supplement: S2 Table — (DOCX) [file ppat.1010820.s005.docx]

Table S2 Sequences of microRNA (miRNA) mimics/ inhibitors and siRNA used in this study.

| Name | Sequence (5’-3’) |
| --- | --- |
| ssc-let-7a mimic | UGAGGUAGUAGGUUGUAUAGUU |
| ssc-let-7a inhibitor | AACUAUACAAUCUACUACCUCA |
| ssc-let-7c mimic | UGAGGUAGUAGGUUGUAUGGUU |
| ssc-let-7c inhibitor | AACCAUACAACCUACUACCUCA |
| ssc-let-7d mimic | CUAUACGACCGUCUGCCUUUCU |
| ssc-let-7d inhibitor | AGAAAGGCAGCAGGUCGUAUAG |
| ssc-let-7e mimic | UGAGGUAUGAGGUUGUAUAGUU |
| ssc-let-7e inhibitor | AACUAUACAACCUCCUACCUCA |
| ssc-let-7f mimic | UGAGGUAGUAGAUUGUAUAGUU |
| ssc-let-7f inhibitor | AACUAUACAAUCUACUACCUCA |
| ssc-let-7g mimic | UGAGGUAGUAGUUUGUACAGUU |
| ssc-let-7g inhibitor | AACUGUACAAACUACUACCUCA |
| ssc-let-7i mimic | UGAGGUAGUAGUAGUUUGUGGU |
| ssc-let-7i inhibitor | AGCACAAACUACUACCUCA |
| NC mimic | UUCUCCGAACGUGUCACGUTT |
| NC inhibitor | CAGUACUUUUGUGUAGUACAA |
| mir-98 mimic | UGAGGUAGUAAGUUGUAUUGUU |
| mir-98 inhibitor | AACAAUACAACUUACUACCUCA |
| SiIL6 | GCCUGGAAGAAGAUGCCAATT |
| siNC | UUCUCCGAACGUGUCACGUTT |
