# Supplementary material for: miRNA let-7 family regulated by NEAT1 and ARID3A/NF-κB inhibits PRRSV-2 replication in vitro and in vivo
Source: PLoS Pathog. 2022 Oct 10;18(10):e1010820. doi: 10.1371/journal.ppat.1010820 (PMC9550049; doi:10.1371/journal.ppat.1010820)
Supplement: S3 Table — (DOCX) [file ppat.1010820.s006.docx]

**Table S3 Sequences of primers used for RT-qPCR in this study.**

| Primer | Sequence(5’-3’) |
| --- | --- |
| Probe-ORF7 | TCCCGGTCCCTTGCCTCTGGA |
| ORF7-F | TAGGTGACTTAGGCACAGT |
| ORF7-R | TAAATATGCCAAATAACAAC |
| IL6-F | AAGTCCAGTCGCCTTCTCCC |
| IL6-R | CCCGTCTCCTTTCTCATTGC |
| β-actin-F | TCATCACCATTGGCATGAG |
| β-actin-R | AGCACTGTGTTGGCGTACAG |
| ssc-miR-210-F | GTGGTAGGCTGTGCGTGT |
| ssc-miR-331-3p-F | TAGCCCCTGGGCCTATCCTAGAACT |
| ssc-miR-27a-F | TGGTAGGTTCACAGTGGCTAAGTTC |
| ssc-let-7a-F | ACACTCCAGCTGGGTGAGGTAGTAGGTTGT |
| ssc-let-7c-F | ACACTCCAGCTGGGTGAGGTAGCAGGTTGT |
| ssc-let-7d-F | ACACTCCAGCTGGGTGAGGTAGTAGGTTGT |
| ssc-let-7e-F | ACACTCCAGCTGGGTGAGGTAGGAGGTTGT |
| ssc-let-7f-F | ACACTCCAGCTGGGTGAGGTAGTAGATT |
| ssc-let-7g-F | ACACTCCAGCTGGGTGAGGTAGTAGTTTGT |
| ssc-let-7i-F | ACACTCCAGCTGGGTGAGGTAGTAGTT |
| ssc-mir-98-F | ACACTCCAGCTGGGTGAGGTAGTAAGTTGT |
| Universal primer-R | TCAACTGGTGTCGTGGAGTCGGC |
| U6-F | GCTTCGGCAGCACATATACT |
| U6-R | TTCACGAATTTGCGTGTCAT |
| ICAM-1-F | CAGTGTTGCCTGTGATGGAAA |
| ICAM-1-R | CTTCAGTCTTGTGCCAGTGAGTCT |
| MCP-1-F | CCAGCAGCAAGTGTCCTAAAG |
| MCP-1-R | TTTTCTTGTCCAGGTGGCTTAT |
| VCAM-1-F | TCTGAATGAAAAGTGAAAAC |
| VCAM-1-R | GACACCTGACTGTAACTGGCT |
| ARID3A-F | GACAGCAACCGACGGGAGGGC |
| ARID3A-R | TTAGGGGCGGGGGTGATGGAG |
| NEAT1-F | CGCCTAAATTGATGTCTGC |
| NEAT1-R | TCTGCTTGGGACTTGGAAC |
| P65-F | CATGCGCTTCCGCTACAAG |
| P65-R | GGTCCCGCTTCTTTACACAC |
