# Supplementary material for: miRNA let-7 family regulated by NEAT1 and ARID3A/NF-κB inhibits PRRSV-2 replication in vitro and in vivo
Source: PLoS Pathog. 2022 Oct 10;18(10):e1010820. doi: 10.1371/journal.ppat.1010820 (PMC9550049; doi:10.1371/journal.ppat.1010820)
Supplement: S4 Table — (DOCX) [file ppat.1010820.s007.docx]

**Table S4 Probe sequences for EMSA.**

| ARID3A-F-BIO-1 | CATTGGTTTTCTTTTAATAAGATCACTTA |
| --- | --- |
| ARID3A-R-BIO-1 | TAAGTGATCTTATTAAAAGAAAACCAATG |
| ARID3A-F-C-1 | CATTGGTTTTCTTTTAATAAGATCACTTA |
| ARID3A-R-C-1 | TAAGTGATCTTATTAAAAGAAAACCAATG |
| ARID3A-F-M-1 | CATTGGTTTTCTTAATTTAAGATCACTTA |
| ARID3A-R-M-1 | TAAGTGATCTTATTAAAAGAAAACCAATG |
| ARID3A-F-BIO-2 | TATTGCATTTAATCATAGATTATG |
| ARID3A-R-BIO-2 | CATAATCTATGATTAAATGCAATA |
| ARID3A-F-C-2 | TATTGCATTTAATCATAGATTATG |
| ARID3A-R-C-2 | CATAATCTATGATTAAATGCAATA |
| ARID3A-F-M-2 | TATTGCATAATTTCATAGATTATG |
| ARID3A-R-M-2 | CATAATCTATGAAATTATGCAATA |
| ARID3A-F-BIO-3 | CTTTCTCCTTTAATCACACAGGAG |
| ARID3A-R-BIO-3 | CTCCTGTGTGATTAAAGGAGAAAG |
| ARID3A-F-C-3 | CTTTCTCCTTTAATCACACAGGAG |
| ARID3A-R-C-3 | CTCCTGTGTGATTAAAGGAGAAAG |
| ARID3A-F-M-3 | CTTTCTCCAAATTACACACAGGAG |
| ARID3A-R-M-3 | CTCCTGTGTGTAATTTGGAGAAAG |
