# Supplementary material for: miRNA let-7 family regulated by NEAT1 and ARID3A/NF-κB inhibits PRRSV-2 replication in vitro and in vivo
Source: PLoS Pathog. 2022 Oct 10;18(10):e1010820. doi: 10.1371/journal.ppat.1010820 (PMC9550049; doi:10.1371/journal.ppat.1010820)
Supplement: S5 Table — (DOCX) [file ppat.1010820.s008.docx]

**Table S5 PCR sequence for CHIP assay.**

| CHIP-1site -F | TCTTTGATCTTCACAGCACTT |
| --- | --- |
| CHIP-1site -R | CAGCACTAAAAGGACAAAACA |
| CHIP-2site -F | TTGTCCTTTTAGTGCTGC |
| CHIP-2site -R | GTATTAGACTCCTTTCATTCAC |
| CHIP-3site -F | GAATGAAAGGAGTCTAATAC |
| CHIP-3site -R | TTGAGTGATGCTTGGATGAT |
